# A Descriptive Whole-Genome Transcriptomics Study in a Stem Cell-Based Tool Predicts Multiple Tissue-Specific Beneficial Potential and Molecular Targets of Carnosic Acid

**DOI:** 10.3390/ijms24098077

**Published:** 2023-04-29

**Authors:** Farhana Ferdousi, Kazunori Sasaki, Satoshi Fukumitsu, Hidetoshi Kuwata, Mitsutoshi Nakajima, Hiroko Isoda

**Affiliations:** 1 Alliance for Research on the Mediterranean and North Africa (ARENA), University of Tsukuba, Tsukuba 305-8572, Japan; ferdousi.farhana.fn@u.tsukuba.ac.jp (F.F.); sasaki-kazu@aist.go.jp (K.S.);; 2 Open Innovation Laboratory for Food and Medicinal Resource Engineering (FoodMed-OIL), National Institute of Advanced Industrial Science and Technology (AIST), Tsukuba 305-0821, Japan; 3 NIPPN Corporation, Tokyo 243-0041, Japan; 4 Tsukuba Life Science Innovation Program (T-LSI), University of Tsukuba, Tsukuba 305-8577, Japan; 5 MED R&D Corporation, Tsukuba 305-8572, Japan; 6 Faculty of Life and Environmental Sciences, University of Tsukuba, Tsukuba 305-8572, Japan

**Keywords:** carnosic acid, human amniotic epithelial stem cell, whole-genome transcriptomics, food functionality, drug discovery, natural compound

## Abstract

Carnosic acid (CA) is a phenolic diterpene widely distributed in herbal plants, rosemary and sage. Although its medicinal properties, such as antioxidant, antimicrobial, and neuroprotective effects, have been well-documented, its relevant biochemical processes and molecular targets have not been fully explored yet. In the present study, we conducted an untargeted whole-genome transcriptomics analysis to investigate CA-induced early biological and molecular events in human amniotic epithelial stem cells (hAESCs) with the aim of exploring its multiple tissue-specific functionalities and potential molecular targets. We found that seven days of CA treatment in hAESCs could induce mesoderm-lineage-specific differentiation. Tissue enrichment analysis revealed that CA significantly enriched lateral plate mesoderm-originated cardiovascular and adipose tissues. Further tissue-specific PPI analysis and kinase and transcription factor enrichment analyses identified potential upstream regulators and molecular targets of CA in a tissue-specific manner. Gene ontology enrichment analyses revealed the metabolic, antioxidant, and antifibrotic activities of CA. Altogether, our comprehensive whole-genome transcriptomics analyses offer a thorough understanding of the possible underlying molecular mechanism of CA.

## 1. Introduction

In high-throughput drug screening, from target identification to preclinical compound evaluation, human pluripotent stem cells have been widely exploited as physiologically relevant in vitro human models. In addition, stem cell-based methods shorten the durations and minimize the attrition rate of new therapeutics development. However, the main obstacles to the widespread use of pluripotent stem cells, including ESCs and iPSCs, for drug screening and toxicity testing include the scarcity of cell resources, invasive extraction techniques, expensive cell reprogramming and maintenance processes, and ethical considerations [1,2,3].

In this regard, perinatal stem cell—human amniotic epithelial stem cells (hAESCs) derived from biological waste products may offer an attractive cell source in drug screening and toxicity testing activities. Since hAESCs are derived from discarded term placenta, they are readily available, require no invasive harvesting approaches, and have few ethical restrictions. Importantly, because of their embryonic origin, hAESCs possess pluripotency and multipotency characteristics [4,5,6]. hAESCs can be differentiated into cells from all three germ layers: bone and fat cells from mesodermal origin, hepatocyte and cholangiocyte from endodermal origin, and neural cells from the ectodermal origin [5,7,8,9,10].

In our earlier studies, we have been examining how natural compounds of plant origin affect the early biological events in hAESCs as part of our ongoing research into exploring the bioactivities and functionalities of these compounds [11,12,13,14,15,16]. We revealed for the first time that distinct natural compounds could trigger targeted differentiation of hAESCs in a lineage-specific manner, such as a flavonol aglycone isorhamnetin could induce hepatic-lineage-specific differentiation [17], an anthocyanin, cyanidin 3-glucoside (CY3G) showed adipose-tissue-specific differentiation [18], whereas a caffeic acid ester, rosmarinic acid, and a caffeoylquinic acid derivative, 3,4,5-Tri-O-Caffeoylquinic acid (TCQA), enhanced neural cell differentiation [12,15]. We observed that the differentiation direction induced by the natural compounds in hAESCs could also predict their known bioactivities. For example, isorhamnetin is well documented to reduce hepatic fibrosis in a variety of in vivo settings [19,20], while CY3G is well-recognized for its capacity to control adipocyte differentiation and have antiobesity and antidiabetic properties [21,22]. Rosmarinic acid has been shown to have neuroprotective benefits in neuroinflammatory and neurodegenerative diseases, which has been attributed to its ability to stimulate neuronal differentiation [23,24,25]. Therefore, in the present study, we aimed to explore the bioactivities of carnosic acid (CA) by examining its effect on early cellular responses in hAESCs.

CA is a phenolic diterpene widely distributed in herbal plant extracts, such as rosemary (*Rosmarinus officinalis*) and sage (*Salvia officinalis*). CA possesses antioxidant, antimicrobial, and neuroprotective properties and, therefore, is increasingly being used in food and nutritional supplements and cosmetics industries [26,27,28,29,30]. Since its first extraction from a Salvia species more than 80 years ago [31], numerous studies have investigated the medicinal applications of CA. However, relevant biochemical processes and molecular targets of CA are yet to be explored. In the present study, we analyzed genome-wide transcriptional responses to CA in hAESCs to explore its multiple tissue-specific functionalities and potential molecular targets.

## 2. Results

### 2.1. Carnosic Acid Significantly Regulated Gene Expression in hAESCs

**Figure 1:**
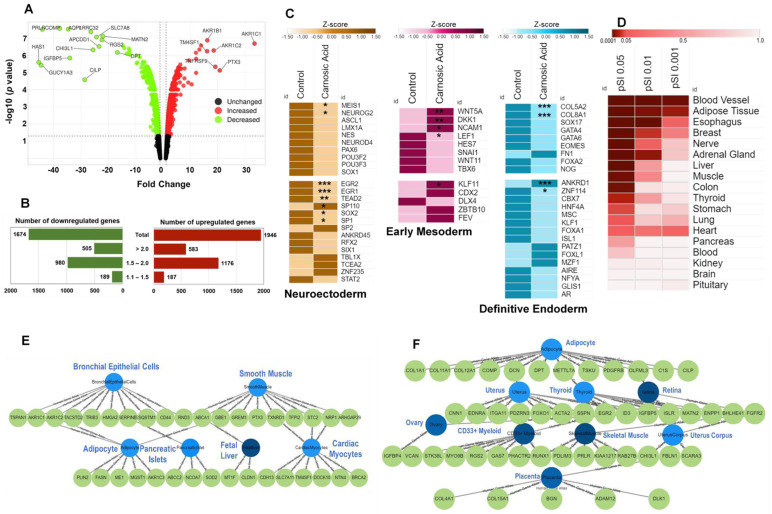
Characterization of gene expression profile in CA-treated hAESCs. (**A**) Volcano plot displaying differentially expressed genes (DEGs) between CA-treated and control hAESCs on day 7. The vertical axis (*y*-axis) corresponds to the −log10 *p* value and the horizontal axis (*x*-axis) displays linear fold change. The red dots represent the upregulated genes; the green dots represent the downregulated genes. The top 20 DEGs with the biggest fold changes are shown in the figure. (**B**) Bar graphs showing the distribution of fold changes. (**C**) Heatmaps showing the expression patterns of the transcription factors specific to three germ layers—neuroectoderm, early mesoderm, and definitive mesoderm. Color code represents the z-score of gene expression values. Darker shade represents upregulation. Significant genes are marked with asterisks; * *p* < 0.05, ** *p* < 0.01, and *** *p* < 0.0001. Heat map was generated on Morpheus tool (https://software.broadinstitute.org/morpheus/ (accessed on 8 March 2023)). (**D**) Heatmap showing the significance and specificity of the expressions of the DEGs across different tissues. Tissue enrichment analysis was conducted using TSEA tool. (**E**) Top enriched tissue types from human gene atlas database based on top 100 significantly upregulated DEGs. (**F**) Top enriched tissue types from human gene atlas database based on top 100 significantly downregulated DEGs. The blue circles in (**E**,**F**) panels refer to significantly enriched tissue or cell types from the human gene atlas database, while the green circles represent the tissue/cell-type-specific DEGs. Color intensity of the blue circles represents the significance of enrichment. The more significant the tissue/cell types, the lighter the blue circles.

A total of 21,449 probe sets were identified on Clariom S Assay Human Array. Compared to control hAESCs, 3620 genes were differentially expressed in CA-treated hAESCs that passed the preliminary quality control threshold (Detected above background (DABG) < 0.05, positive vs. negative area under the curve (AUC) > 0.7, and one-way ANOVA *p* value < 0.05). Among them, 1946 genes were upregulated and 1674 were downregulated. Significantly expressed genes between CA-treated and control hAESCs are displayed in the volcano plot (Figure 1A). The *y*-axis corresponds to the −log10 *p* value and the *x*-axis corresponds to linear fold change. The red dots represent the upregulated genes; while the green dots represent the downregulated genes. The top 20 differentially expressed genes (DEGs) with the largest fold changes (FC) are pointed out on the volcano plot. The top downregulated DEG with the highest fold change was hyaluronan synthase 1 (*HAS1*, FC = −45.45), while the top upregulated DEG was aldo-keto reductase family 1, member C1 (*AKR1C1*, FC = 33.45). The top 10 up- and downregulated DEGs and their biological functions are listed in Table 1 and Table 2, respectively.

The distribution of fold changes in up- and downregulated genes is shown in the butterfly graph (Figure 1B). Most of the significant genes showed FC > 1.5. A total of 1095 genes passed our final filter criteria of fold change > 2 (in linear space) and *p* value < 0.05 (one-way between-subject ANOVA). After removing duplicate and no symbol genes, a total of 1088 genes (upregulated DEGs = 583 and downregulated DEGs = 505) were considered as DEGs and were included in subsequent enrichment analyses.

### 2.2. Carnosic Acid Biased hAESCs toward Mesodermal Lineage Progression

Our previous study reported that the undifferentiated hAESCs, both 2D and 3D plated cells, could highly express several stem cell markers, such as SSEA4, TRA 1-60, TRA 1-81, EPCAM, E–cadherin, and Lectin, and have multilineage differentiation potential [15].

Therefore, we next examined the effects of CA treatment on the expressions of three germ-layer-specific markers to explore whether CA could direct the targeted differentiation of hAESCs in a lineage-specific manner. We found that most of the neuroectoderm and definitive endoderm-specific markers showed decreased expression in CA-treated cells compared to control cells (Figure 1C). Among them, neuroectoderm-specific markers Meis homeobox 1 (*MEIS1*) and neurogenin 2 (*NEUROG2*) and endoderm-specific markers collagen type V, alpha 2 (*COL5A2*) and collagen type VIII, alpha 1 (*COL8A1*) were significantly downregulated. Additionally, several neuroectoderm and endoderm-specific transcription factors (TF) were significantly downregulated, such as early growth response 1 (*EGR1*), early growth response 2 (*EGR2*), TEA domain family member 2 (*TEAD2*), SP110 nuclear body protein (*SP110*), SRY box 2 (*SOX2*), Sp1 transcription factor (*SP1*), ankyrin repeat domain 1 (*ANKRD1*), and zinc finger protein 114 (*ZNF114*).

On the other hand, early mesoderm-specific markers were significantly upregulated in CA-treated cells compared to control cells, such as wingless-type MMTV integration site family, member 5A (*WNT5A*), Dickkopf WNT signaling pathway inhibitor 1 (*DKK1*), neural cell adhesion molecule 1 (*NCAM1*), and lymphoid enhancer-binding factor 1 (*LEF1*). Additionally, a well-known mesoderm-specific TF, Kruppel-like factor 11 (*KLF11*), was significantly upregulated (Figure 1C).

Next, we performed tissue-specific enrichment analysis (Figure 1D). Among the 1084 DEGs, 1034 exist in the tissue expression dataset [32]. Mesoderm-derived blood vessels and adipose tissue were the most significantly enriched tissues in all Specificity Index thresholds (pSI) varying from 0.05 to 0.0001. In addition, the esophagus, breast, nerve, adrenal gland, and heart tissues were also significantly enriched in all pSI thresholds. On the other hand, the liver, muscle, colon, and stomach were enriched in the low pSI threshold (pSI 0.05).

Finally, to confirm the tissue specificity in CA-treated hAESCs, we limited the enrichment analysis to the top 100 up- and downregulated DEGs. Adipocytes, bronchial epithelial cells, cardiac myocytes, and smooth muscle are among the tissues most enriched by the top 100 upregulated DEGs (Figure 1E), while the placenta, ovary, uterus, uterus corpus are among the tissues most enriched by the top 100 downregulated DEGs (Figure 1F). These findings suggest that seven days of CA treatment could induce, at least in part, mesoderm lineage-specific differentiation in hAESCs.

### 2.3. Carnosic Acid Regulated a Wide Range of Biological and Molecular Events in hAESCs

Natural compounds are widely documented for their ability to influence cellular activity and regulate biological and molecular processes in stem cells, enabling their functional prediction. Therefore, we performed a detailed gene ontology (GO) analysis to explore the potential bioactivities of CA.

**Figure 2:**
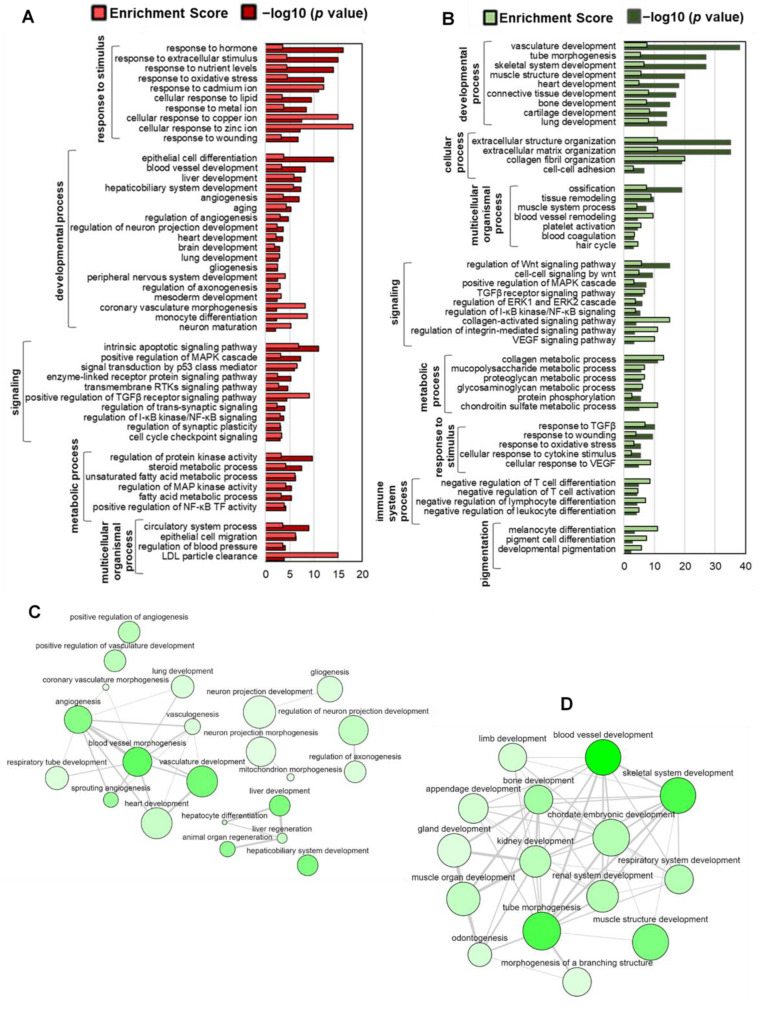
Biological process gene ontology (GOBP) enrichment analysis of CA-treated hAESCs. Top enriched GOBP terms were selected based on *p* value from hypergeometric distribution. (**A**) Top enriched GOBP terms by the upregulated DEGs. (**B**) Top enriched GOBP terms by the downregulated DEGs. (**C**) Interactions among the ‘developmental process’-associated GO terms by the upregulated DEGs. (**D**) Interactions among ‘developmental process’-associated GO terms by the downregulated DEGs. The color of the discs indicates significance, and their size is proportional to the number of genes in the category. The thickness of grey lines indicates the degree of simRel semantic similarity across categories.

We performed GO biological process (GOBP) analyses for up- and downregulated DEGS (FC > 2) separately (Figure 2A,B). First, we identified significantly enriched parent GOBP terms by the DEGs (Figure S1). Top enriched parent GOBP terms by the upregulated DEGs include response to stimulus (GO:0050896), developmental process (GO:0032502), signaling (GO:0023052), metabolic process (GO:0008152), and multicellular organismal process (GO:0032501).

Response to stimulus included response to hormones, nutrient levels—especially, lipids, oxidative stress, and several ions. Significantly enriched developmental processes included epithelial cell and monocyte differentiation, aging process, and development of blood vessels, liver, heart, brain, and coronary vasculature (Figure 2A). Interactions among the developmental process-related GOBP terms reveal that blood vessel morphogenesis, angiogenesis, and heart development share the most similar gene sets (Figure 2C). Other top enriched GOBP terms included apoptotic signaling pathway, steroid and fatty acid metabolic process, MAPK cascade, TGFβ receptor signaling pathway, and NFκB TF activity. Some important upregulated BPs also included blood pressure regulation, axonogenesis, gliogenesis, and LDL particle clearance (Figure 2A).

Top enriched parent GOBP terms by the downregulated DEGs included developmental process, cellular process (GO:0009987), multicellular organismal process, signaling, metabolic process, response to stimulus, immune system process (GO:0002376), and pigmentation (GO:0043473) (Figure S1).

Significantly downregulated developmental processes included the development of vasculature, skeletal system, and muscle structure (Figure 2B). GOBP interaction analysis revealed that bone and skeletal system development had the most similar gene sets (Figure 2D). Significantly enriched cellular processes by the downregulated DEGs included extracellular matrix and structure organization and collagen fibril organization. Top downregulated BP terms also included platelet activation, blood coagulation, hair cycle process, and differentiation of melanocyte and pigment cells (Figure 2B).

**Figure 3:**
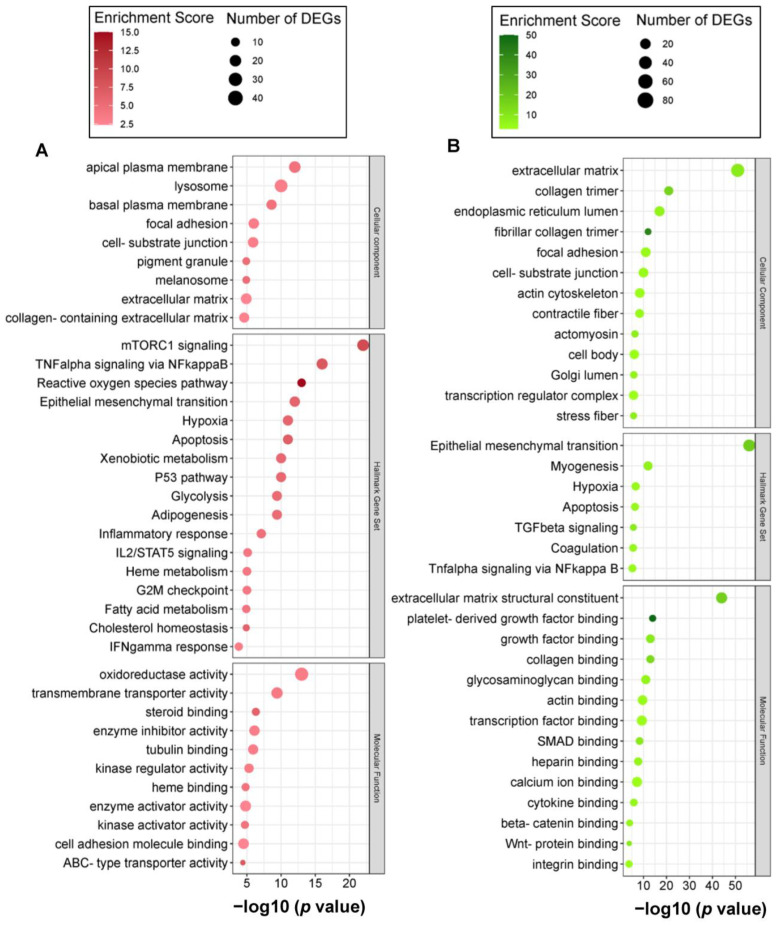
Gene set overrepresentation analysis of CA-treated hAESCs. Bubble plots showing significantly enriched cellular components, Hallmark gene sets, and molecular functions by (**A**) upregulated DEGs, and (**B**) downregulated DEGs. The *x*-axis represents –log10 (*p* value), color code represents enrichment score, and bubble size represents number of DEGs in each term. Significant gene sets were identified based on *p* values from hypergeometric distribution.

Top upregulated cellular component GO (GOCC) terms included apical plasma membrane and lysosome and molecular function (GOMF) terms included oxidoreductase activity, transmembrane transporter activity, steroid binding, tubulin binding, kinase regulator activity, and heme binding (Figure 3A). On the other hand, top downregulated GOCC and GOMF terms included extracellular matrix, collagen binding, collagen fibril, focal adhesion, and growth factor binding—specifically platelet-derived growth factor binding (Figure 3B).

The top enriched Hallmark gene sets mirrored the GO enrichment analysis results. The upregulated Hallmark gene sets included mTORC1 signaling, TNFα signaling via NFκB, reactive oxygen species pathway, adipogenesis, glycolysis, and fatty acid metabolism (Figure 3A), whereas downregulated gene sets included epithelial–mesenchymal transition, hypoxia, apoptosis, TGFβ signaling, coagulation, Tnfα signaling via NFκB (Figure 3B).

### 2.4. Carnosic-Acid-Regulated Germ-Layer-Specific Pathways in hAESCs

Next, we performed pathway enrichment analysis. Significantly enriched KEGG pathways included the MAPK signaling pathway, insulin resistance, TNF signaling pathway, lung fibrosis, TGFβ receptor signaling, pre-NOTCH Transcription and Translation, signaling by NOTCH, canonical and noncanonical TGFβ signaling.

**Figure 4:**
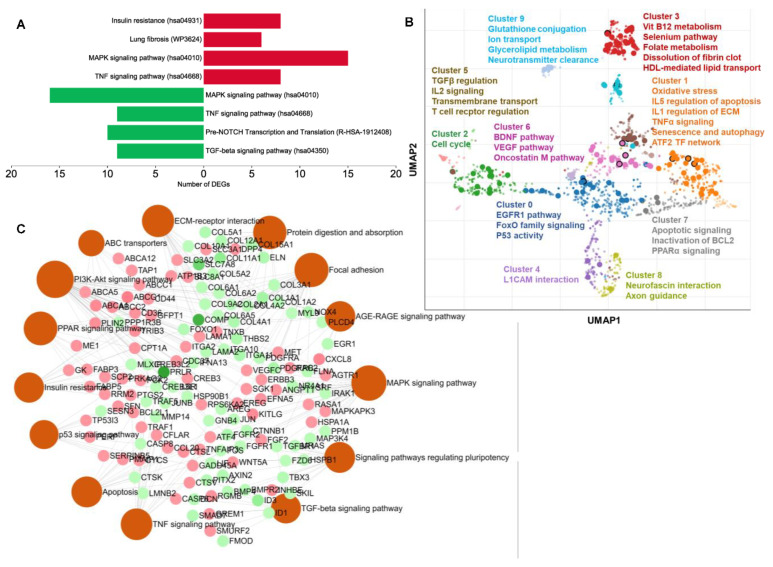
Pathway enrichment analysis of CA-treated hAESCs. (**A**) Enrichment pattern of essential signaling pathways for germ layer differentiation in CA-treated hAESCs. The *x*-axis represents number of DEGs in CA-treated hAESCs. Red and green bars represent the number of up- and downregulated DEGS, respectively. (**B**) Scatterplot showing similar pathway gene set clusters based on BioPlanet 2019 gene set library. Term frequency–inverse document frequency (TF–IDF) values were computed for the gene set corresponding to each term, and the UMAP dimensionality reduction technique was applied to the resulting values. The terms are plotted based on the first two UMAP dimensions. Terms are colored by automatically identified clusters computed with the Leiden algorithm applied to the TF–IDF values. (**C**) Interaction network showing top enriched KEGG pathways and related DEGs.

We found that the MAPK signaling pathway, insulin resistance, and lung fibrosis-related genes were upregulated (Figure 4A). These pathways are reported to be regulated during mesodermal differentiation. On the other hand, ectoderm- and endodermal-specific TGFβ and NOTCH signaling pathways were found to be downregulated (Figure 4A).

To identify functionally related pathway clusters in CA-treated cells, we applied the UMAP dimensionality reduction technique. The terms were plotted based on the first two UMAP dimensions (Figure 4B). This pathway enrichment analysis included both up- and downregulated DEGs (FC > 2) in order to identify the most predominant clusters and pathway terms. We found that metabolic and inflammatory pathways were the most highlighted terms in the scatter plot (Figure 4B).

Next, we built an interaction network of the KEGG pathways that further revealed that pluripotency, focal adhesion, TGFβ, and extracellular matrix-related DEGs were downregulated. In contrast, PPAR signaling, insulin resistance, and PI3K–Akt signaling pathway-related DEGs were upregulated (Figure 4C). In Supplementary File S1, there are details for Figure 4C.These results add to the evidence that CA may mediate pathways specific to the mesoderm in hAESCs. CA may also have potential metabolic functions as well as strong antifibrotic activity.

### 2.5. Protein–Protein Interaction Network Topography in Carnosic Acid-Treated hAESCs

**Figure 5:**
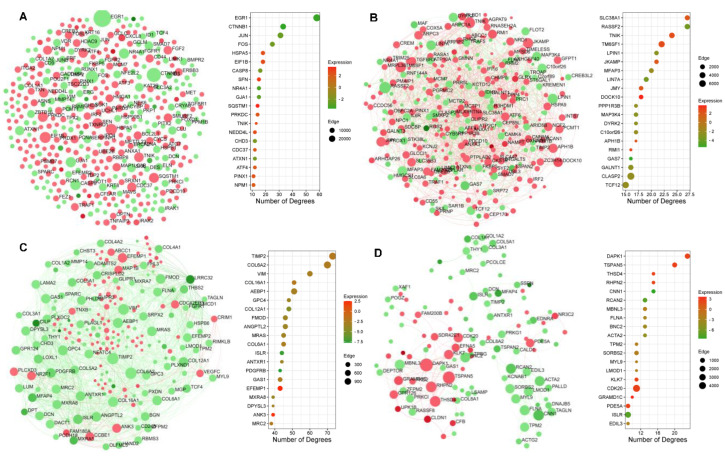
Generic and Tissue-Specific Zero-Order PPI network by the DEGS in CA-treated hAESCs. (**A**) Generic PPI interaction network showing key hub nodes in CA-treated hAECs. Tissue-specific gene coexpression networks (**B**) Whole-blood-specific, (**C**) Liver-specific, and (**D**) Adipose-tissue-specific. Each node represents proteins and edges indicating known interactions (InnateDB Interactome database) between two connecting proteins. Red and green nodes denote up- and downregulated genes, respectively. Bubble plots showing corresponding top 20 hub genes (seeds).

We conducted DEG-based protein–protein interaction (PPI) network analysis to identify the interacting genes with similar attributes and functions (Figure 5). All DEGs (FC > 2) were used to construct these undirected zero-order networks, with nodes representing genes and edges representing gene–gene associations. Green and red nodes represent down and upregulated DEGs, respectively. Edges are also shown in red and green color to represent the regulation. A list of all hub nodes is provided in Supplementary File S2.

First, we performed a generic PPI analysis to identify the hub nodes, authenticated as the top nodes with the highest degree, in CA-treated hAESCs (Figure 5A). A total of 426 seeds with 728 edges were identified. *EGR1* was the top hub node with the highest degree (degree = 58, betweenness = 27,492.56, FC = −3.49), followed by *CTNNB1* (degree = 33, betweenness = 14,852.74, FC = −3.59).

Next, we constructed several tissue-specific gene coexpression networks, including whole blood (Figure 5B), liver (Figure 5C), and adipose-tissue-specific (Figure 5D) networks. In addition, the coronary-artery-, heart-atrium-, ventricle-, and skeletal-muscle-specific networks are provided in Figure S2.

The whole-blood-specific network identified 335 seeds with 1044 edges, while *RASSF2* (degree = 27, betweenness = 2974.49, FC = −3.69) and *SLC38A1* (degree = 27, betweenness = 2376.06, FC = 2.09) were the top hub nodes.

Liver-specific gene coexpression network identified 213 seeds with 1537 edges. Liver-specific top seeds included *TIMP2* (Degree = 73, Betweenness = 1131.28), *COL6A2* (Degree = 70, Betweenness = 1136.17), *VIM* (Degree = 60, Betweenness = 491.28), *AEBP1* (Degree = 51, Betweenness = 723.88), *COL16A1* (Degree = 51, Betweenness = 230.04), and *COL12A1* (Degree = 48, Betweenness = 299.51). These seed nodes had downregulated expression in CA-treated conditions compared to the control.

The adipose-tissue-specific gene coexpression network identified 128 seeds with 297 edges (Figure 5D). Top seeds included *DAPK1* (Degree = 20, Betweenness = 1107.06), *TSPAN5* (Degree = 15, Betweenness = 398.03), *CNN1* (Degree = 15, Betweenness = 139.47), *RHPN2* (Degree = 15, Betweenness = 121.25), *THSD4* (Degree = 13, Betweenness = 725.3), and *RCAN2* (Degree = 13, Betweenness = 414.69). *CNN1* and *RCAN2* had downregulated expression in CA-treated cells compared to the control cells.

### 2.6. Significantly Enriched Transcription Factors and Kinases in Carnosic-Acid-Treated hAESCs

TFs and kinases are critical modulators of important signaling pathways, and their actions are required for many essential cellular functions. Therefore, we performed TF and kinase enrichment analyses to identify upstream regulators likely responsible for the observed patterns in genome-wide gene expression in CA-treated hAESCs.

**Figure 6:**
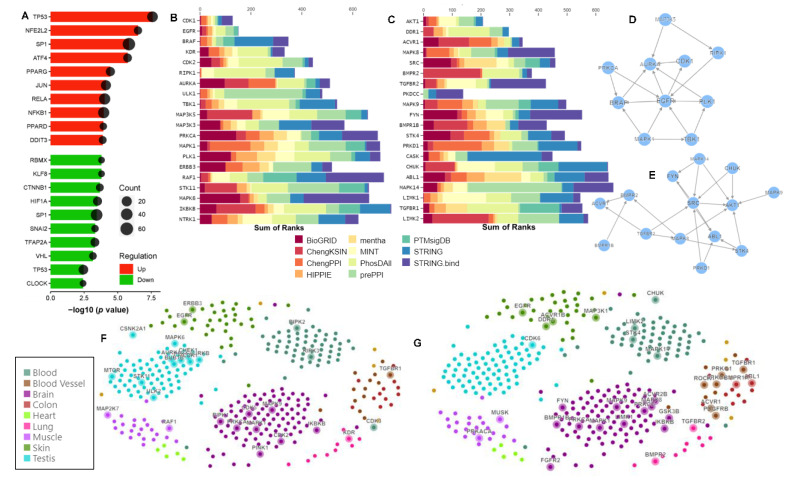
TF and kinase enrichment analyses of CA-treated hAESCs. (**A**) Bar graph showing significantly enriched transcription factors from TRRUST database. Red and green bars represent enrichment by the up- and downregulated DEGs, respectively. Bubble size represents the number of DEGs regulated by each TF in CA-treated hAESCs. Stacked Bar graphs showing upstream kinases whose putative substrates are overrepresented in (**B**) upregulated DEGs and (**C**) downregulated DEGs. The bar represents the mean rank of kinases based on multiple libraries (color-coded by library). Kinase co-regulatory networks constructed from kinase–kinase interactions between top-ranked kinases, (**D**) by upregulated DEGs and (**E**) by downregulated DEGs. Directed edges indicate interactions supported by kinase–substrate evidence; undirected edges indicate protein–protein interaction or unspecified interaction evidence only. Human kinome regulatory networks of (**F**) upregulated DEGs and (**G**) downregulated DEGs by applying Weighted Gene Coexpression Network Analysis (WGCNA) to the Genotype–Tissue Expression (GTEx) dataset. Each color-coded WGCNA module eigengene was correlated to GTEx tissue. Tissue-specific kinases were identified based on integrated mean rank.

Significantly enriched TFs by the upregulated DEGs included TP53 (*n* = 40, *p* value = 2.58 × 10^−8^), NFE2L2 (*n* = 10, *p* value = 3.11 × 10^−7^), SP1 (*n* = 78, *p* value = 1.46 × 10^−6^), ATF4 (*n* = 14, *p* value = 1.85 × 10^−6^), and PPARG (*n* = 18, *p* value = 3.52 × 10^−5^), JUN (*n* = 30, *p* value = 7.54 × 10^−5^), RELA (*n* = 50, *p* value = 9.36 × 10^−5^), NFKB1 (*n* = 50, *p* value = 1.11 × 10^−4^), PPARD (*n* = 6, *p* value = 1.18 × 10^−4^), and DDIT3 (*n* = 8, *p* value = 1.32 × 10^−4^). On the other hand, significantly enriched TFs by the downregulated DEGs included KLF8 (*n* = 5, *p* value = 1.61 × 10^−4^), RBMX (*n* = 5, *p* value = 1.61 × 10^−4^), CTNNB1 (*n* = 8, *p* value = 2.14 × 10^−4^), HIF1A (*n* = 17, *p* value = 3.21 × 10^−4^), SP1 (*n* = 60, *p* value = 3.72 × 10^−4^), SNAI2 (*n* = 6, *p* value = 4.88 × 10^−4^), TFAP2A (*n* = 15, *p* value = 5.01 × 10^−4^), and VHL (*n* = 7, *p* value = 7.02 × 10^−4^) (Figure 6A).

Significantly enriched upstream kinases whose putative substrates are overrepresented in the upregulated DEGs included CDK1, EGFR, BRAF, KDR, CDK2, RIPK1, AURKA, ULK1, TBK1, MAP3K5, MAP3K3, PRKCA, MAPK1, and PLK1 (Figure 6B). Significantly downregulated kinases included AKT1, DDR1, ACVR1, BMPR2, TGFBR2, PKDCC, and FYN (Figure 6C). The kinases were ranked based on the Mean Rank score across different kinase libraries [33].

Next, we constructed kinase co-regulatory networks from kinase–kinase interactions between top-ranked kinases (Figure 6D,E). Directed edges indicate interactions supported by kinase–substrate evidence. EGFR was found to be the top regulatory kinase by the upregulated DEGs (Figure 6D), whereas AKT1 was the top regulatory kinase by the downregulated DEGs (Figure 6E).

Further, we constructed human kinome regulatory networks of up- and downregulated DEGs by applying Weighted Gene Coexpression Network Analysis (WGCNA) to the Genotype–Tissue Expression (GTEx) [34] dataset (Figure 6F,G). We identified several tissue-specific kinase enrichments. Blood-vessel-specific TGFBR1; blood-specific RIPK1 and RIPK2; skin-specific EGFR and ERBB3; muscle-specific RAF1 and MAP2K7; and brain-specific MAPK1, MAPK9, CDK2, CDK5, PRKCA, IKBKB, PINK1, and RIPK1 were enriched (based on integrated mean rank) by the upregulated DEGs. On the other hand, blood-vessel-specific TGFBR1, PDGFRB, ACVR1, and ROCK1; blood-specific CHUK, LIMK2, STK4, and MAPK14; skin-specific EGFR, ACVR1B, and MAP3K1; muscle-specific MUSK, PRKAKA, lung-specific TGFBR2, and BMPR2; and brain-specific MAPK1, MAPK9, LIMK1, PRKCE, IKBKB, GSK3B, FYN, and BMPR1B were enriched by the downregulated DEGs.

### 2.7. Chemical and Disease Perturbation Enrichment Analyses by the DEGs in Carnosic Acid-Treated hAESCs

**Figure 7:**
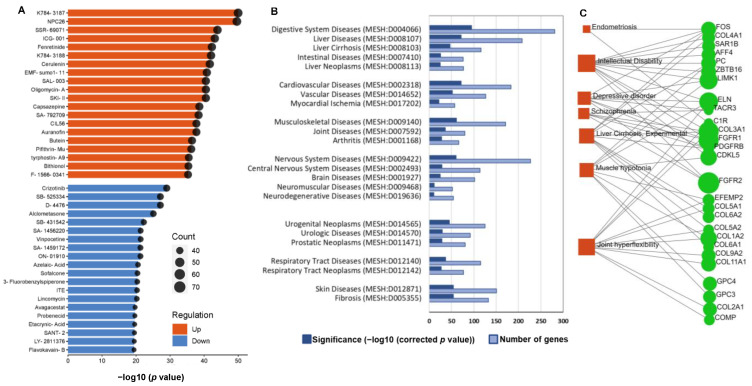
Chemical perturbations and disease–gene association enrichment analyses. (**A**) Bar graph showing the list of chemicals that show significant similarity with the DEGs in CA-treated hAESCs (LINCS L1000 Chemical Perturbation Consensus library). Orange and blue bars represent the enrichment by the up- and downregulated DEGs, respectively. Bubble size represents the number of DEGs. (**B**) Significantly enriched disease terms (Comparative Toxicogenomics Database) by the differentially expressed genes (fold change > 2, both up- and downregulated DEGs). (**C**) Network analysis showing enriched gene–disease association by downregulated DEGs (DisGeNET database).

Wide-ranging biological and therapeutic applications, particularly drug development, can be drawn from inferring a relationship between chemical perturbations and their unique transcriptional response. Therefore, we performed enrichment analyses of chemical and disease perturbations to predict similar perturbation responses in CA-treated hAESCs (Figure 7). Chemical and disease perturbations were conducted using the LINCS L1000 Chemical Perturbation Consensus library [35] and DisGeNET database [36], respectively. These resources have collected gene expression profiles for thousands of perturbagens and have been successfully employed for various studies.

We performed chemical perturbation enrichment analyses separately for up- and downregulated DEGs to identify the chemicals that showed similar gene expression patterns (Figure 7A).

Upregulated DEGs enriched K784-3187 (Overlapped genes = 71, Adjusted *p* value = 1.12 × 10^−46^), NPC26 (Overlapped genes = 70, Adjusted *p* value = 1.29 × 10^−46^), SSR-69071 (Overlapped genes = 66, Adjusted *p* value = 4.34 × 10^−41^), ICG-001 (Overlapped genes = 64, Adjusted *p* value = 1.96 × 10^−40^), fenretinide (Overlapped genes = 64, Adjusted *p* value = 1.15 × 10^−39^), K784-3188 (Overlapped genes = 64, Adjusted *p* value = 1.68 × 10^−39^), cerulenin (Overlapped genes = 64, Adjusted *p* value = 3.29 × 10^−39^), SKI-II (Overlapped genes = 62, Adjusted *p* value = 3.03 × 10^−38^), oligomycin A (Overlapped genes = 62, Adjusted *p* value = 3.03 × 10^−38^), and capsazepine (Overlapped genes = 61, Adjusted *p* value = 2.28 × 10^−36^).

Downregulated DEGs enriched Crizotinib (Overlapped genes = 49, Adjusted *p* value = 6.37 × 10^−26^), D-4476 (Overlapped genes = 47, Adjusted *p* value = 1.26 × 10^−24^), SB-525334 (Overlapped genes = 47, Adjusted *p* value = 1.26 × 10^−24^), alclometasone (Overlapped genes = 45, Adjusted *p* value = 7.94 × 10^−23^), vinpocetine (Overlapped genes = 41, Adjusted *p* value = 1.98 × 10^−19^), and azelaic acid (Overlapped genes = 40, Adjusted *p* value = 1.04 × 10^−18^).

Next, we investigated significantly enriched disease terms from the comparative toxicogenomics database [37] using both up- and downregulated DEGs (FC > 2). Enriched diseases are the MEDIC terms, a modified subset of descriptors from the MeSH and OMIM databases, that are statistically enriched among our input genes. Significantly enriched disease terms included the digestive system—specifically liver diseases, cardiovascular, musculoskeletal, nervous system, fibrosis, and skin diseases (Figure 7B). These findings suggest that CA may have potential health benefits for these diseases.

Gene–disease association network analysis using the downregulated DEGs showed that liver cirrhosis, depressive disorder, muscle hypotonia, and endometriosis-associated genes were downregulated in CA-treated hAESCs (Figure 7C).

## 3. Discussion

In the present study, we have performed an integrated whole-genome transcriptome analysis of CA-treated hAESCs. Our data indicate that CA has multiple tissue-specific health benefits.

Every year, a large number of natural compounds-derived small molecules become available for drug screening and biological research. Whilst there have been significant technological advancements, the rate of new medicines being discovered is remarkably slow. The time lag between a compound’s preclinical validation and successful clinical application is a major hindrance to drug discovery. The translational inefficiency of new target compounds is caused by the unpredictability of the currently employed in vitro cellular models, where the critical components of drug–biology interaction are lost, and the complexity of the in vivo milieu. In this regard, hAESC, a perinatal stem cell, offers an attractive and physiologically relevant in vitro human model for drug screening and toxicity assessment [16].

We previously showed that different natural compounds could induce lineage-specific differentiation of multipotent hAESCs [12,15,17,18]. It became evident from our previous findings that the bioactivities of natural compounds may indeed be extensively anticipated from the enriched cell types by the DEGs. In the present study, we found that CA significantly upregulated mesoderm-specific markers while downregulated ectoderm and endoderm-specific markers (Figure 1C). Subsequently, tissue enrichment analysis showed that the upregulated DEGs significantly enriched the lateral plate mesoderm-originated tissues, such as the cardiovascular system (blood vessels, heart), adipose tissue, and smooth muscle (Figure 1D,E). On the other hand, downregulated DEGs significantly enriched paraxial mesoderm-specific tissues, such as the musculoskeletal system (bone, skeletal muscle, and cartilage) and intermediate mesoderm-specific tissues, such as the reproductive system (uterus and ovary) (Figure 1F and Figure 2B,D). Vasculature smooth muscle and development were found to be at the center of upregulated developmental GO (Figure 1E and Figure 2A,C). Recent studies showed that lateral plate mesoderm and neural crest contribute to the smooth muscle components of the blood vasculature [38,39], which explains the enrichment of the brain and peripheral nervous system enrichment by the upregulated DEGs (Figure 2A).

In line with the adipocyte differentiation potential, a substantial metabolic function of CA can also be predicted from the GO and pathway enrichment analyses (Figure 2A, Figure 3A and Figure 4A,B). Upregulated DEGs significantly enriched carbohydrate and lipid metabolism-related GOBP terms—steroid and fatty acid metabolic process (Figure 2A) and hallmark gene sets—glycolysis and cholesterol homeostasis (Figure 3A). Additionally, several signal transduction pathways that directly modulate the metabolic reprogramming of cells to enable cell survival and proliferation were also significantly upregulated in CA-treated hAESCs, which included PI3K–Akt, MAPK, EGFR1, and mTORC1 signaling pathways [40,41]. Additionally, EGFR and several MAP kinases were found to be upstream regulators of upregulated DEGs in CA-treated hAESCs (Figure 6B,D). These findings suggest the multitarget potential of CA through modulating complex interacting biochemical processes.

A strong antifibrotic potential of CA can also be predicted from the enrichment and PPI analyses. We found that CA significantly downregulated connective tissue development, collagen fibril organization, collagen metabolic process, collagen binding, platelet-derived growth factor binding, and integrin binding (Figure 2B and Figure 3B). Additionally, the TGFβ pathway, the master regulator of fibrosis [42], was also significantly downregulated (Figure 3B and Figure 4A,C). Focal adhesion and extracellular matrix organization-related genes were also significantly downregulated in CA-treated hAESCs (Figure 3B and Figure 4C). Moreover, kinase enrichment analysis identified TGFBR2 and BMPs as the top interacting upstream regulators of downregulated DEGs in CA-treated conditions (Figure 6E). The antifibrotic properties of CA were further supported by identifying many liver fibrosis-specific seed genes in PPI analysis, including *TIMP2*, *COL6A1*, and *COL6A2* (Figure 5C) [43,44].

TF enrichment analysis shows that TP53 was enriched by both up- and downregulated DEGs (Figure 6A). The p53-dependent transcriptional program initiates a series of events resulting in cell cycle arrest, senescence, or apoptosis in a context-dependent manner [45,46]. Other top-enriched TFs by the upregulated DEGs included NFE2L2 (or, Nrf2)—a transcriptional activator that binds to the antioxidant response element and orchestrates the adaptive response to an oxidative challenge [47], NFκB—a pivotal mediator of inflammatory responses [48], RELA—an NFκB subunit, PPARG—a key regulator of adipogenesis and glucose and fatty acid metabolism [49], and ATF4—a modulator of amino acid metabolism and oxidative stress response [50]. We have also identified a number of tissue-specific upstream kinases (Figure 6F,G), which may serve as the molecular targets of CA. For example, CA-enriched blood-vessel-specific TGFBR1 kinase, which is an important regulator of cardiovascular health and tumor vasculature [51,52,53]. Particularly, blood-vessel-specific PDGFRB and ROCK1 were enriched by the downregulated genes by CA treatment. It has been suggested that PDGFRB and ROCK1 inhibition promotes anticancer, antiangiogenic, antihypertensive, and antiatherosclerosis effects [54,55]. Additionally, RAF1 activation in muscle leads to MAPK-signaling-pathway-dependent muscle cell proliferation [56]. Several brain-specific kinases that mediate energy metabolism (MAPKs) and neuroinflammation (IKBKB) are also found to be enriched in CA-treated cells. Indeed, CA and CA-rich plant extracts, such as rosmarinic acid, have been extensively researched for their neuroprotective properties [28,57,58,59,60].

Further, chemical perturbation enrichment analysis (Figure 7A) shows that CA significantly upregulated gene sets similar to several chemicals/drugs, such as NPC26—a mitochondrion-interfering compound that kills human colorectal cancer cells via activating AMPK signaling [61], ICG 001—a Wnt/β catenin pathway inhibitor [62], fenretinide—a synthetic retinoid derivative that acts as an antioxidant and chemopreventive agent [63], and cerulenin—an antifungal and antilipemic drug [64]. On the other hand, downregulated DEGs enriched similar downregulated gene sets of crizotinib—a receptor tyrosine kinases inhibitor [65], vinpocetine—a neuroprotective natural compound [66], and alclometasone—a topical corticosteroid [67].

Finally, gene–disease association analysis summarizes the beneficial potential of CA in cardiovascular, metabolic, liver fibrosis, and depressive disorders (Figure 7B,C). Indeed, a number of studies have documented the preventive effects of CA in liver fibrosis [68,69,70], depressive disorders [71,72,73], and metabolic diseases [74,75,76].

Our comprehensive whole-genome transcriptomics analyses offer a thorough understanding of the possible underlying molecular mechanism of CA. We have identified multiple tissue-specific potential upstream regulators and molecular targets of CA. However, our study is not immune to the inherent limitations of microarray investigations, such as low accuracy, specificity, and precision. Further functional cellular assays and in vivo evaluation are warranted to validate our findings.

## 4. Materials and Methods

### 4.1. hAESCs Isolation and Cell Culture Maintenance

We received the cells from ‘The Tsukuba Human Tissue Biobank Center (THB)’ established at the University of Tsukuba Hospital in 2013 [77]. Procedures for hAEC isolation and culture have been described in detail elsewhere [15,78]. Briefly, hAESCs were isolated from the term placenta. Firstly, the amnion was aseptically separated from the chorion and then washed with 200 mL of Hank’s Basic Salt Solution—Calcium and Magnesium Free (CMF–HBSS). The amnion was cut into smaller pieces using surgical scissors and placed in 50 mL conical tubes. A total of 20 mL pre-digestion buffer containing CMF–HBSS and 0.5 mM EGTA was added to the tubes. The amnion was gently rocked in the solution for 30 s and then transferred to new 50 mL conical tubes. Again, 20 mL pre-digestion buffer was added to the tubes and was incubated at 37 °C for 10 min. Next, the pre-digestion buffer was discarded, and 20 mL of 0.05% trypsin–EDTA was added to the tubes, incubated for 40 min at 37 °C, and then placed on ice. Next, Dulbecco’s Modified Eagle Medium (DMEM, 10% FBS, 1% Penn–Strep) was added to the trypsin digest and centrifuged at 200× *g* for 10 min at 4 °C. After discarding the supernatant, the pellet was resuspended in 10 mL of DMEM and filtered through a 100 μm filter.

Cells were maintained in Placental Epithelial Cell Basal Medium (PromoCell, Cat. # C-26140) and were constantly monitored with media change every 2–4 days. Cells were first cultured in 2D plates, as described previously [15].

### 4.2. Preparation of 3D Spheroid Formation and Cell Treatment

The cells were cultured in 3D culture plates (Elplasia™, Kuraray Co., Ltd., Cat # RB 500 400 NA 24, Chiyoda City, Japan) coated with Lipidure™ solution (NOF Corporation, Cat. # CMS206; 400 μL, Tokyo, Japan) (50 mg in 10 mL absolute ethanol) as described previously. hAESC spheroids were formed by seeding 1 × 10^6^ cells into each well of the 24-well plates. Cells were seeded in the Placental Basal Epithelial Cell Medium and cultured for 24 h.

After 72 h of initial culture, cells were treated with 20 μM of CA (purchased from Fujifilm Wako Pure Chemicals, Tokyo, Japan, Code No. 039-22151). Control samples were maintained in the Placental medium. The medium was changed every 48 h, three times during the seven days treatment duration. Finally, we collected the treatment and control samples from the one-week culture.

### 4.3. RNA Extraction and Quantification

According to the manufacturer’s guide, the RNA was extracted using the Isogen kit (Nippon Gene Co. Ltd., Tokyo, Japan). Then, RNA quantity and quality were determined using the NanoDrop 2000 spectrophotometer (ThermoScientific, Waltham, MA, USA).

### 4.4. DNA Microarray Analysis

DNA microarray analysis was conducted on control and CA-treated hAESC samples using the GeneChip WT PLUS Reagent Kit (ThermoFisher Scientific, Waltham, MA, USA) and GeneChip™ Hybridization, Wash and Stain Kit (ThermoFisher Scientific, Waltham, MA, USA) following the manufacturer’s instructions. In brief, complementary DNA (cDNA) was synthesized from 100 ng of RNA solutions. cRNA was synthesized from in vitro transcription of cDNA and then purified and reverse transcribed. Finally, single-stranded cDNA (ss-cDNA) was synthesized, purified, fragmented, and labeled following the manufacturer’s instructions.

Cartridge Array Hybridization was performed using the Clariom S array (Human; ThermoFisher Scientific, Waltham, MA, USA) on the GeneChip™ Fluidics Station (ThermoFisher Scientific, Waltham, MA, USA). Scanning was performed using GeneChip Scanner (ThermoFisher Scientific, Waltham, MA, USA).

### 4.5. Microarray Data Processing and Quality Control

The raw image data obtained after scanning were analyzed using the Transcriptome Analysis Console (TAC) software (version 4.0.2, ThermoFisher Scientific, Waltham, MA, USA). The raw data were normalized following the signal space transformation robust multi-chip analysis (SST–RMA) algorithm. Further, gene-level analysis was performed using the Limma Bioconductor package, which is included with TAC 4.0.2. For differential expression analysis, a one-way ANOVA followed by an empirical Bayes correction was performed. The detected above background (DABG) cutoff was set to 0.05. The positive vs. negative area under the curve (AUC) value was set at greater than or equal to 0.7. Finally, genes that passed the filter criteria of *p* value < 0.05 (one-way between-subject ANOVA) and fold change > 2 (in linear space) were considered as differentially expressed genes (DEGs).

### 4.6. Microarray Data Analysis

The volcano plot was created using the VolcaNoseR web application (accessed on 19 February 2023) [79]. Heatmaps were drawn using the Broad Institute’s Morpheus online tool (https://software.broadinstitute.org/morpheus/) (accessed on 8 March 2023). 

The tissue-specific enrichment was determined using the Tissue-Specific Expression Analysis (TSEA) web tool (version 1.0, http://genetics.wustl.edu/jdlab/tsea/, accessed on 20 February 2023) [32]. The gene expression data used in TSEA were collected from the Genotype–Tissue Expression (GTEx) project [34]. Tissue enrichment was expressed as the ‘Specificity Index’ thresholds (pSI), where pSI < 0.0001 denotes the most specific genes enriched in a particular tissue. The significance of tissue enrichment was calculated by Fisher’s Exact test.

Gene ontology (GO) and pathway enrichment analyses (overrepresentation analysis) were performed using the web-based tool Metascape (v3.5.20230101, http://metascape.org, accessed on 19 February 2023) [80]. Significant gene sets were identified based on *p* values from hypergeometric distribution. *p* values are provided in negative logarithmic based ten (“−log 10”). Therefore, a higher *p* value indicates the less chance the observed enrichment is due to randomness. Interactions among GO terms were visualized using the REVIGO tool (http://revigo.irb.hr/, accessed on 21 February 2023) [81]. Disc color indicates significance, while the size is proportional to the number of genes in the category. The thickness of grey lines indicates simRel [82] semantic similarity between categories. The spatial arrangements of discs also reflect the grouping of categories by semantic similarity.

The scatterplot for the clusters of functionally similar pathway gene sets was created using the Enrichr Appyter visualization tool (https://maayanlab.cloud/Enrichr/, accessed on 20 February 2023) [83]. NCATS BioPlanet 2019 library was used for gene set cluster identification [84]. Term frequency–inverse document frequency (TF–IDF) values were computed for the gene set corresponding to each term, and the UMAP dimensionality reduction technique was applied to the resulting values. The terms are plotted based on the first two UMAP dimensions. Terms are colored by automatically identified clusters computed with the Leiden algorithm applied to the TF–IDF values.

Protein–protein interaction (PPI) networks were built on the NetworkAnalyst tool (version 3.0) [85]. A generic PPI network was built using the InnateDB Interactome database [86] and the tissue-specific gene coexpression networks were created using the database for tissue/cancer-specific biological networks (TCSBN) [87].

Transcription factor enrichment analysis was performed on Enrichr using the TRRUST database [88]. Upstream kinases were identified using the Kinase Enrichment Analysis version 3 web tool [33]. The top 20 kinases were selected based on integrated rankings of kinases across libraries (Integrated mean rank score). Bar charts are color-coded by kinase libraries. Kinase co-regulatory networks for the top 10 kinases were constructed from kinase–kinase interactions. Directed edges indicate interactions supported by kinase–substrate evidence; undirected edges indicate protein–protein interaction or unspecified interaction evidence. Human kinome regulatory networks were constructed by applying Weighted Gene Coexpression Network Analysis (WGCNA) to the GTEx [34] dataset.

Chemical perturbation analysis was performed on the Enrichr tool using the LINCS L1000 database [35]. Curated gene–disease association data were retrieved from the comparative toxicogenomics database (CTD) [37]. The significance of disease enrichment was calculated using the hypergeometric distribution followed by Bonferroni adjustment for multiple testing. The PPI network for gene–disease associations (downregulated DEGs) was created on the NetworkAnalyst tool using the DisGeNET database [36].

## 5. Conclusions

In conclusion, our comprehensive whole-genome transcriptomics analyses revealed multiple tissue-specific biological and molecular functions of CA. We have also identified potential upstream regulators and molecular targets of CA in a tissue-specific manner. However, further functional cellular assays and in vivo evaluation are warranted to validate our findings.

## Figures and Tables

**Table 1:** Top 10 upregulated genes and their functions.

Gene Symbol	Description	Fold Change	*p* Value	Biological Functions
*AKR1C1*	aldo-keto reductase family 1, member C1	33.45	2.14 × 10^−7^	bile acid and bile salt transport and metabolic process, cholesterol homeostasis, Metabolism of fat-soluble vitamins, lipids and lipoproteins, and xenobiotics by cytochrome P450
*PTX3*	pentraxin 3, long	20.74	7.75 × 10^−6^	extracellular matrix organization, innate immune response, negative regulation by host of viral exo-alpha-sialidase activity,
*TNFRSF9*	tumor necrosis factor receptor superfamily, member 9	19.12	4.78 × 10^−6^	negative regulation of IL10 and IL12 production, TNFR2 non-canonical NFkB pathway
*AKR1C2*	aldo-keto reductase family 1, member C2	18.53	5.38 × 10^−7^	G protein-coupled receptor signaling pathway, positive regulation of protein kinase B signaling, Bile acid and bile salt metabolism
*AKR1B1*	aldo-keto reductase family 1, member B1 (aldose reductase)	16.18	1.38 × 10^−7^	C21-steroid hormone biosynthetic process, negative regulation of apoptotic process, positive regulation of JAK–STAT cascade, positive regulation of smooth muscle cell proliferation
*TM4SF1*	transmembrane 4 L six family member 1	16.11	5.96 × 10^−7^	plasma membrane component
*SLC7A11*	solute carrier family 7 (anionic amino acid transporter light chain, xc- system), member 11	13.89	2.78 × 10^−7^	brain development, adult locomotory behavior, cellular response to oxidative stress
*ABI3BP*	ABI family, member 3 (NESH) binding protein	13.29	4.78 × 10^−7^	defense response to tumor cell, extracellular matrix organization, negative regulation of cell proliferation, negative regulation of connective tissue replacement involved in inflammatory response wound healing, positive regulation of cardiocyte differentiation, gene expression decreases in aging skin
*ANKRD1*	ankyrin repeat domain 1 (cardiac muscle)	12.1	1.63 × 10^−6^	cardiac muscle tissue morphogenesis, myoblast differentiation, cellular response to IL1, TGFβ, and TNF stimulus, Regulation of lipid metabolism by PPARα
*ABCG1*	ATP binding cassette subfamily G member 1	12.1	1.08 × 10^−5^	amyloid precursor protein catabolic process, positive regulation of Aβ formation (suppress), cholesterol efflux, cellular response to HDL particle stimulus, negative regulation of macrophage-derived foam cell differentiation

**Table 2:** Top 10 downregulated genes and their functions.

Gene Symbol	Description	Fold Change	*p* Value	Biological Functions
*HAS1S1*	hyaluronan synthase 1	−45.45	2.64 × 10^−6^	A major component of extracellular matrix that regulates cell adhesion, migration, and differentiation. It is expressed in mesenchymal cells, such as astrocytes in the CNS and fibroblasts in heart, negative regulation of fibroblast migration, cellular response to platelet-derived growth factor stimulus. Stroke induces the expression of HAS1
*GUCY1A3*	guanylate cyclase 1, soluble, alpha 3	−44.58	3.9 × 10^−6^	a key enzyme in the nitric oxide/cGMP signaling pathway, inhibits platelet aggregation upon stimulation with nitric oxide
*PRLR*	prolactin receptor	−44.08	3.24 × 10^−8^	Prolactin signaling pathway, activation of JAK–STAT cascade, PI3K–Akt signaling pathway, leukemia inhibitory factor signaling pathway, highly expressed in depression (rat model, brain), in breast and cervical cancer cells
*COMP*	cartilage oligomeric matrix protein	−37.75	2.81 × 10^−8^	blood coagulation, Focal adhesion, collagen fibril organization, chondrocyte proliferation, BMP signaling pathway, PI3K–Akt signaling pathway, upregulates in cardiac fibrosis and osteoarthritis
*AQP1*	aquaporin 1 (Colton blood group)	−34.66	3.15 × 10^−8^	cellular response to cAMP and to hypoxia, O_2_/CO_2_ exchange in erythrocytes, induces angiotensin II-induced cardiac hypertrophy, increases the risk of myocardial infarction
*IGFBP5*	Insulin-like growth factor binding protein 5	−34.08	1.48 × 10^−6^	Promotes fibrosis and increases the risk of cardiovascular disease, causes abnormal curvature and thinning of the hair shaft, induces IL6-mediated ROS production, and causes premature cell senescence
*CILP*	cartilage intermediate layer protein, nucleotide pyrophosphohydrolase	−28.63	2.7 × 10^−5^	cellular response to TGFβ, negative regulation of insulin-like growth factor receptor signaling pathway, CILP combines with TGFβ or IGF1 to regulate the ECM synthesis in cartilage and promotes degeneration and aging progress in intervertebral discs (IVDs) (but CILP inhibits cardiac fibrosis).
*LRRC32*	Leucine-rich repeat containing 32	−26.39	4.46 × 10^−8^	TGFβ receptor signaling pathway, negative regulation of activated T cell proliferation
*CHI3L1*	chitinase 3-like 1 (cartilage glycoprotein-39)	−25.56	4.97 × 10^−7^	It plays a major role in tissue injury, inflammation, tissue repair, and remodeling responses and has been strongly associated with diseases including asthma, arthritis, sepsis, diabetes, liver fibrosis, coronary artery disease, cancer invasion, and metastasis.
*SLC7A8*	solute carrier family 7 (amino acid transporter light chain, L system), member 8	−24.35	8.9 × 10^−8^	amino acid transmembrane transport

## Data Availability

The supporting data of this article can be found within this paper and in Supplementary Materials. The microarray data have been deposited in the NCBI GEO database and are publicly available (Accession number: GSE228408; https://www.ncbi.nlm.nih.gov/geo/query/acc.cgi?acc=GSE228408 (accessed on 29 March 2023)).

## Supplementary Materials

The following supporting information can be downloaded at: https://www.mdpi.com/article/10.3390/ijms24098077/s1.
