# Supplementary material for: A Descriptive Whole-Genome Transcriptomics Study in a Stem Cell-Based Tool Predicts Multiple Tissue-Specific Beneficial Potential and Molecular Targets of Carnosic Acid
Source: Int J Mol Sci. 2023 Apr 29;24(9):8077. doi: 10.3390/ijms24098077 (PMC10179098; doi:10.3390/ijms24098077)
Supplement: Supplementary file 1 [file ijms-24-08077-s001.zip › Figure S1.pdf]

A

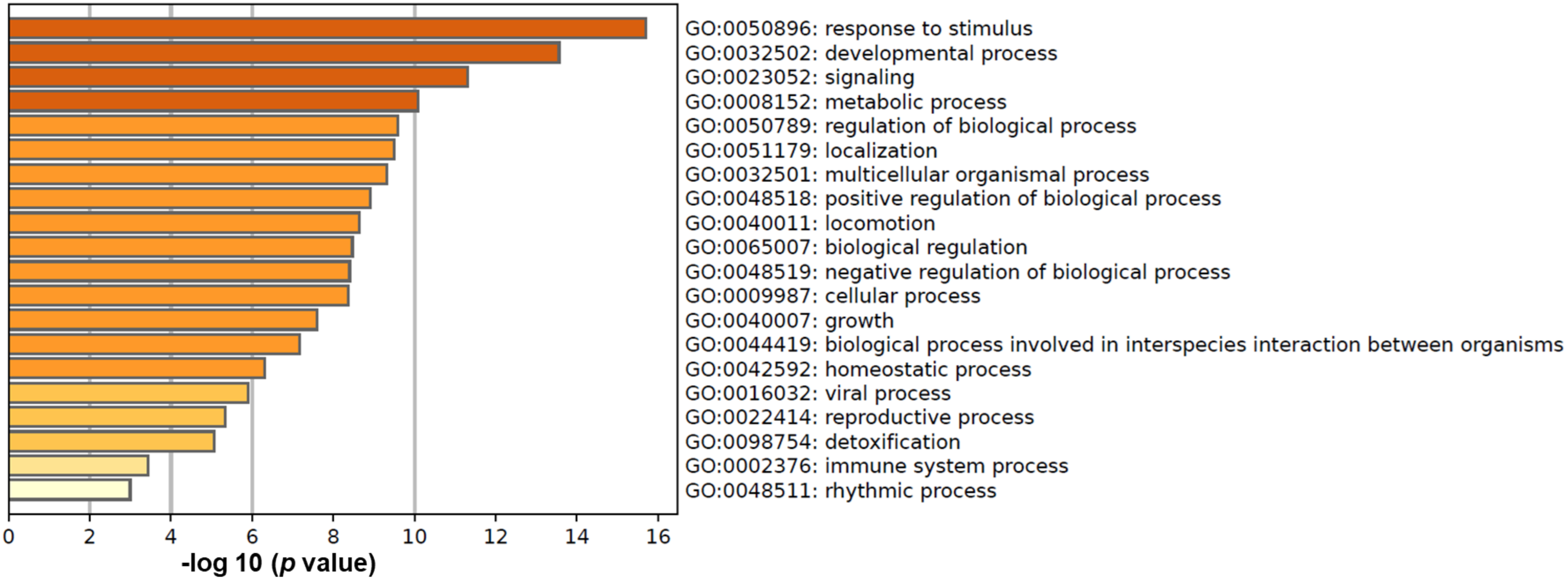

Supplementary Figure S1: (A) Significantly enriched parent GOBP terms by upregulated DEGs, (B) Downregulated DEGs

B

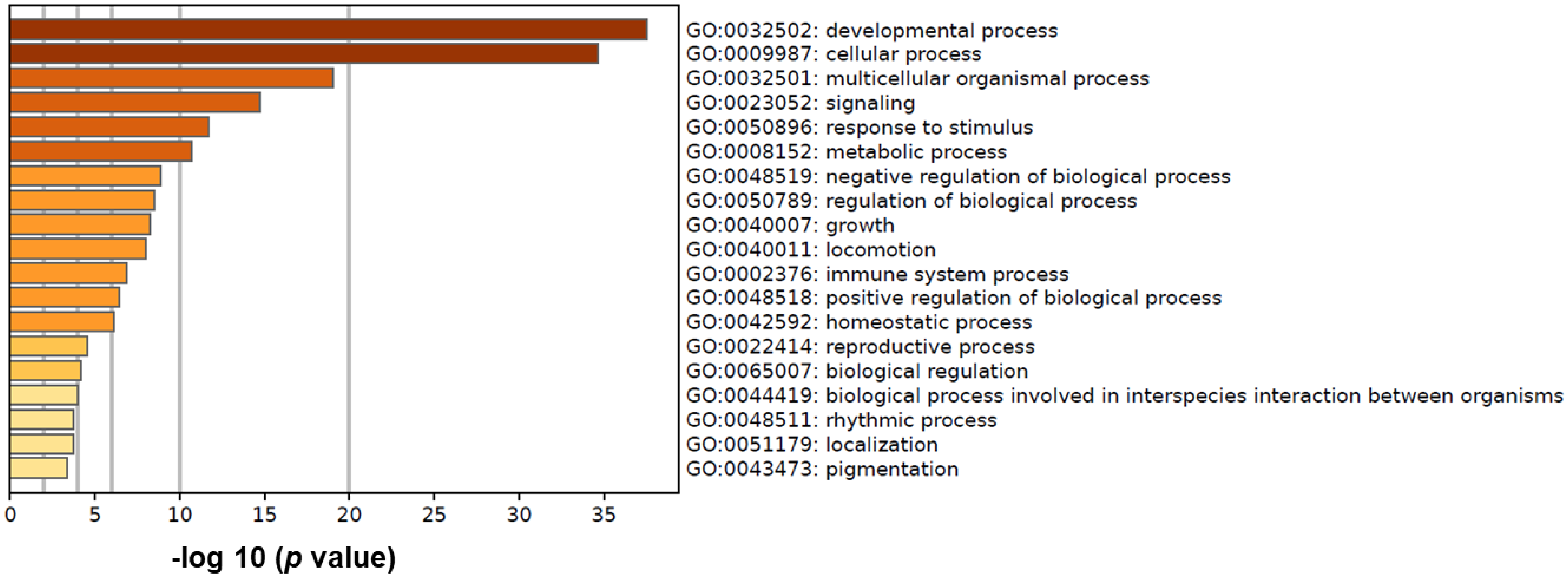

Supplementary Figure S1: (B) Significantly enriched parent GOBP terms by downregulated DEGs
