# Supplementary material for: A Descriptive Whole-Genome Transcriptomics Study in a Stem Cell-Based Tool Predicts Multiple Tissue-Specific Beneficial Potential and Molecular Targets of Carnosic Acid
Source: Int J Mol Sci. 2023 Apr 29;24(9):8077. doi: 10.3390/ijms24098077 (PMC10179098; doi:10.3390/ijms24098077)
Supplement: Supplementary file 1 [file ijms-24-08077-s001.zip › Figure S2.pdf]

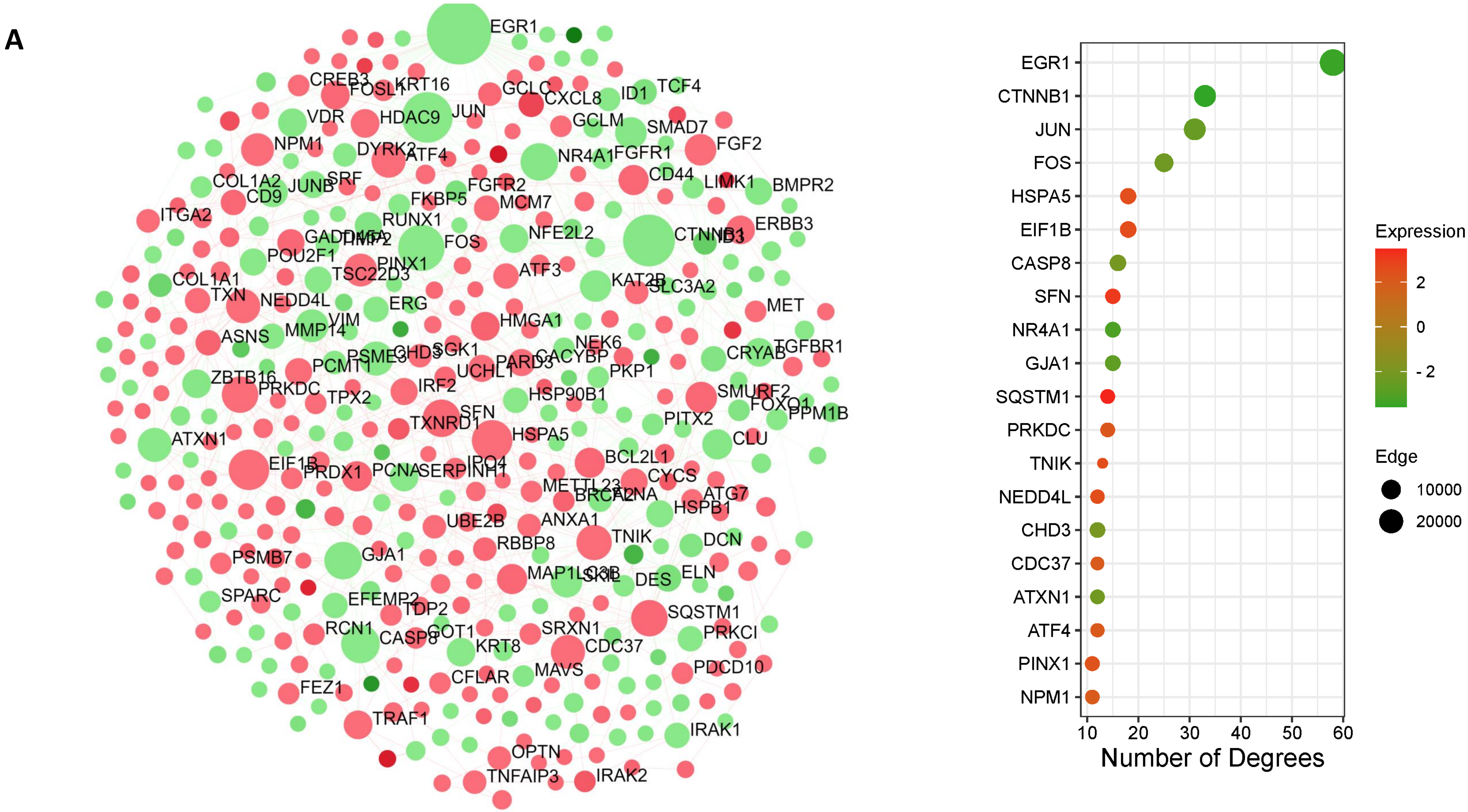
A
EGR1
KRT16
GCLC
CXCL8
ID1
TCF4
VDR
HDAC9
JUN
GCLM
SMAD7
NPM1
NR4A1
FGFR1
FGF2
CD44
BMPR2
JUNB
SRF
CD9
FGFR2
FKBP5
MCM7
ITGA2
RUNX1
ERBB3
POU2F1
FOS
NFE2L2
PINX1
TSC22D3
ATF3
COL1A1
TXN
NEDD4L
ERG
MET
MMP14
ASNS
HMGA1
NEK6
CRYAB
TGFBR1
PCMT1
UCHL1
PKP1
ZBTB16
PRKDC
TPX2
IRF2
HSP90B1
PITX2
ATXN1
TXNRD1
SFN
HSPA5
CLU
BCL2L1
CYCS
PCNA
ATG7
HSPB1
UBE2B
ANXA1
TNIK
DCN
RBBP8
PSMB7
GJA1
DES
ELN
SPARC
SQSTM1
RCN1
SRXN1
KRT8
CDC37
PRKCI
MAVS
PDCD10
CFLAR
FEZ1
TRAF1
IRAK1
OPTN
TNFAIP3
IRAK2
EGR1
CTNNB1
JUN
FOS
HSPA5
EIF1B
CASP8
SFN
NR4A1
GJA1
SQSTM1
PRKDC
TNIK
NEDD4L
CHD3
CDC37
ATXN1
ATF4
PINX1
NPM1
10
20
30
40
50
60
Number of Degrees
Expression
2
0
- 2
Edge
10000
20000

B

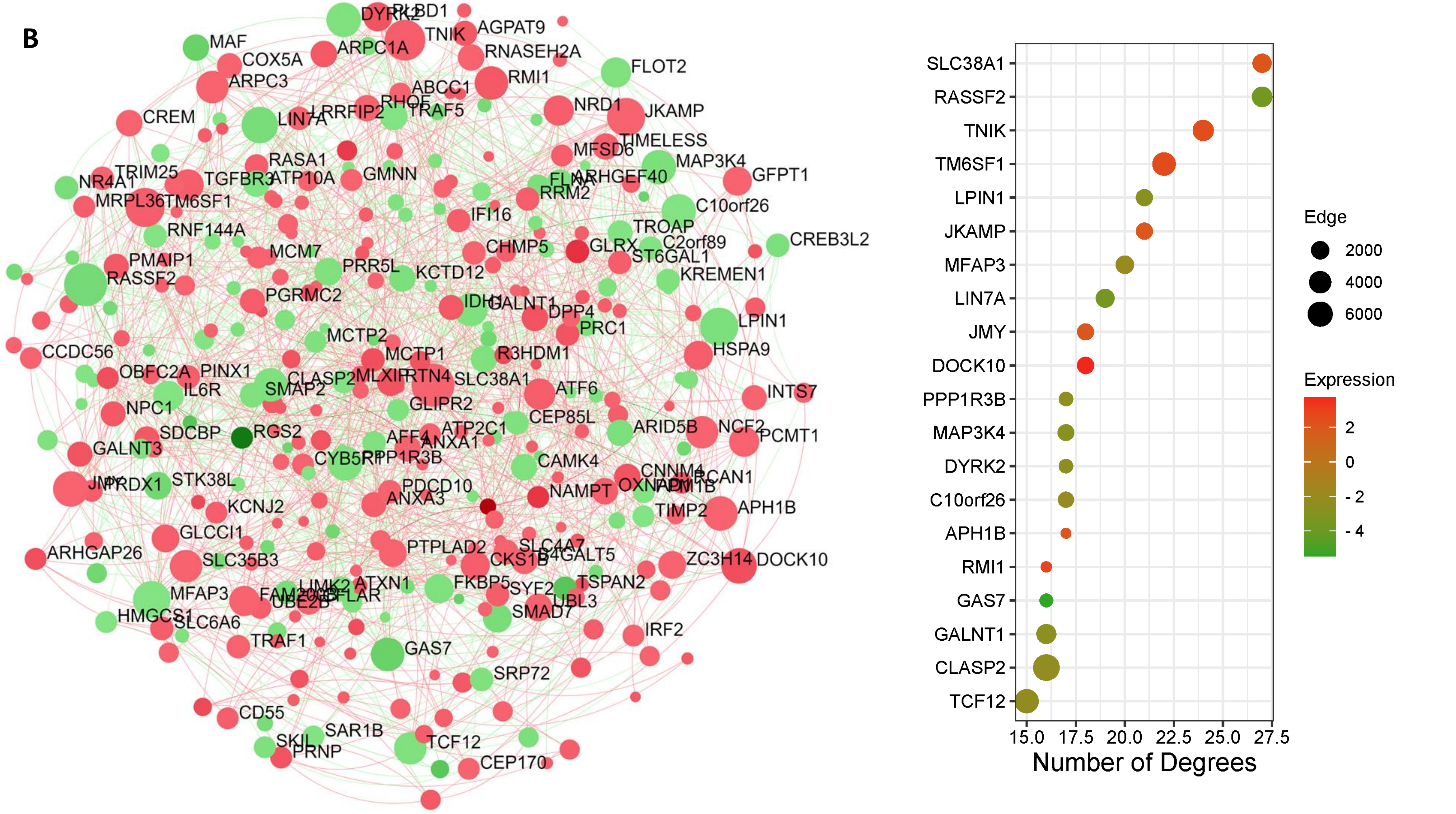

SLC38A1
RASSF2
TNIK
TM6SF1
LPIN1
JKAMP
MFAP3
LIN7A
JMY
DOCK10
PPP1R3B
MAP3K4
DYRK2
C10orf26
APH1B
RMI1
GAS7
GALNT1
CLASP2
TCF12
15.0
17.5
20.0
22.5
25.0
27.5
Number of Degrees
Edge
2000
4000
6000
Expression
2
0
- 2
- 4

C

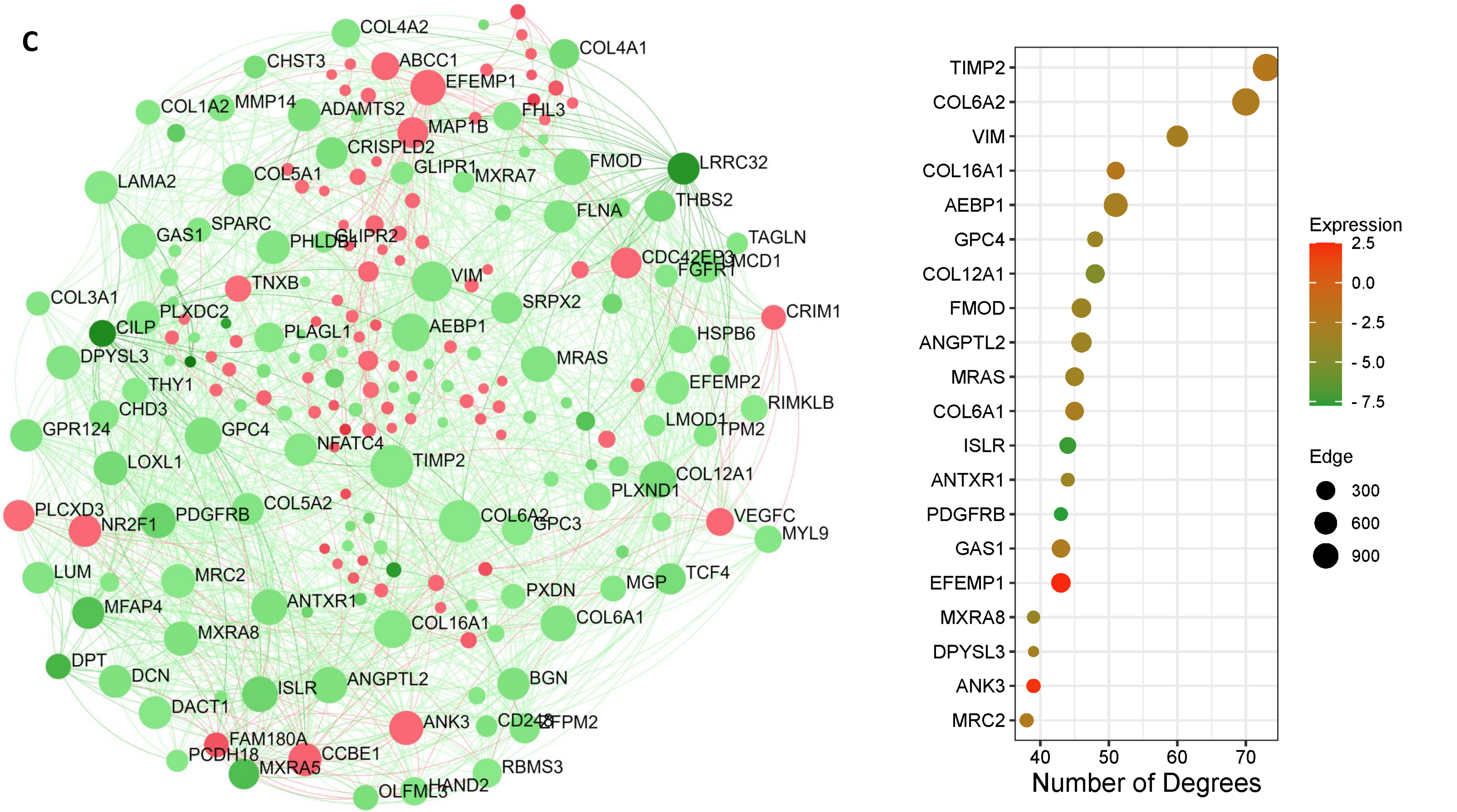

COL4A2
COL4A1
CHST3
ABCC1
EFEMP1
COL1A2
MMP14
ADAMTS2
FHL3
MAP1B
CRISPLD2
GLIPR1
MXRA7
FMOD
LRRC32
LAMA2
COL5A1
THBS2
FLNA
SPARC
GAS1
PHLDB1
GLIPR2
TAGLN
CDC42EP3
LMCD1
FGFR1
TNXB
VIM
COL3A1
SRPX2
CRIM1
PLXDC2
CILP
PLAGL1
AEBP1
HSPB6
DPYSL3
MRAS
THY1
EFEMP2
CHD3
RIMKLB
GPR124
GPC4
LMOD1
TPM2
NFATC4
LOXL1
TIMP2
COL12A1
PLXND1
PLCXD3
NR2F1
PDGFRB
COL5A2
COL6A2
GPC3
VEGFC
MYL9
LUM
MRC2
TCF4
MGP
PXDN
MFAP4
ANTXR1
COL16A1
COL6A1
MXRA8
DPT
DCN
ISLR
ANGPTL2
BGN
DACT1
ANK3
CD248
ZFPM2
FAM180A
CCBE1
PCDH18
MXRA5
RBMS3
OLFML3
HAND2
TIMP2
COL6A2
VIM
COL16A1
AEBP1
GPC4
COL12A1
FMOD
ANGPTL2
MRAS
COL6A1
ISLR
ANTXR1
PDGFRB
GAS1
EFEMP1
MXRA8
DPYSL3
ANK3
MRC2
40
50
60
70
Number of Degrees
Expression
2.5
0.0
- 2.5
- 5.0
- 7.5
Edge
300
600
900

D

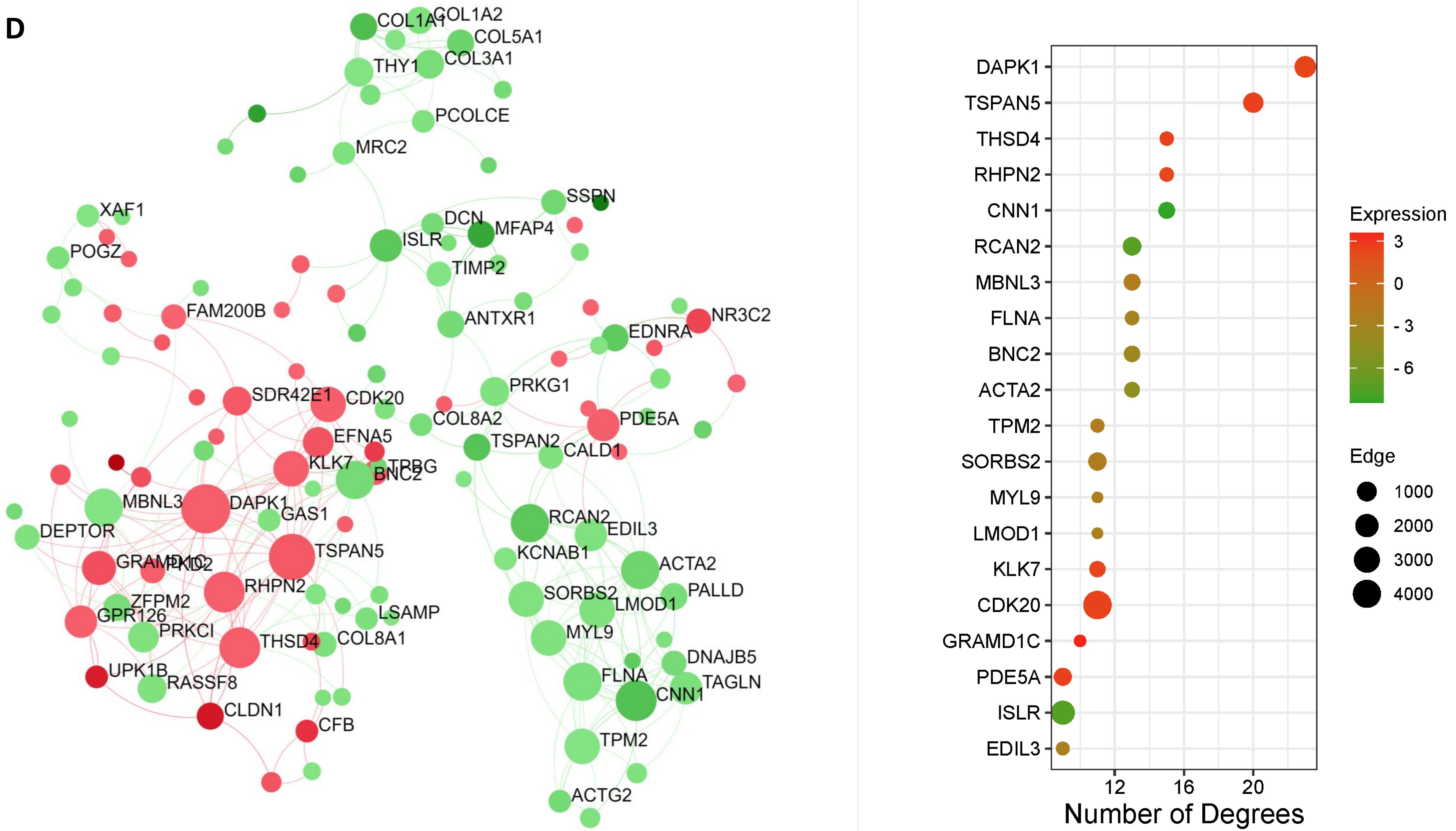
COL1A1
COL1A2
COL5A1
THY1
COL3A1
PCOLCE
MRC2
SSPN
XAF1
DCN
MFAP4
ISLR
POGZ
TIMP2
FAM200B
ANTXR1
NR3C2
EDNRA
PRKG1
SDR42E1
CDK20
COL8A2
PDE5A
EFNA5
TSPAN2
CALD1
KLK7
TPBG
BNC2
MBNL3
DAPK1
GAS1
RCAN2
DEPTOR
EDIL3
TSPAN5
KCNAB1
GRAMD1C
PKD2
ACTA2
RHPN2
PALLD
ZFPM2
SORBS2
GPR126
LMOD1
LSAMP
PRKCI
MYL9
THSD4
COL8A1
UPK1B
DNAJB5
RASSF8
FLNA
TAGLN
CNN1
CLDN1
CFB
TPM2
ACTG2
DAPK1
TSPAN5
THSD4
RHPN2
CNN1
RCAN2
MBNL3
FLNA
BNC2
ACTA2
TPM2
SORBS2
MYL9
LMOD1
KLK7
CDK20
GRAMD1C
PDE5A
ISLR
EDIL3
12
16
20
Number of Degrees
Expression
3
0
- 3
- 6
Edge
1000
2000
3000
4000

E

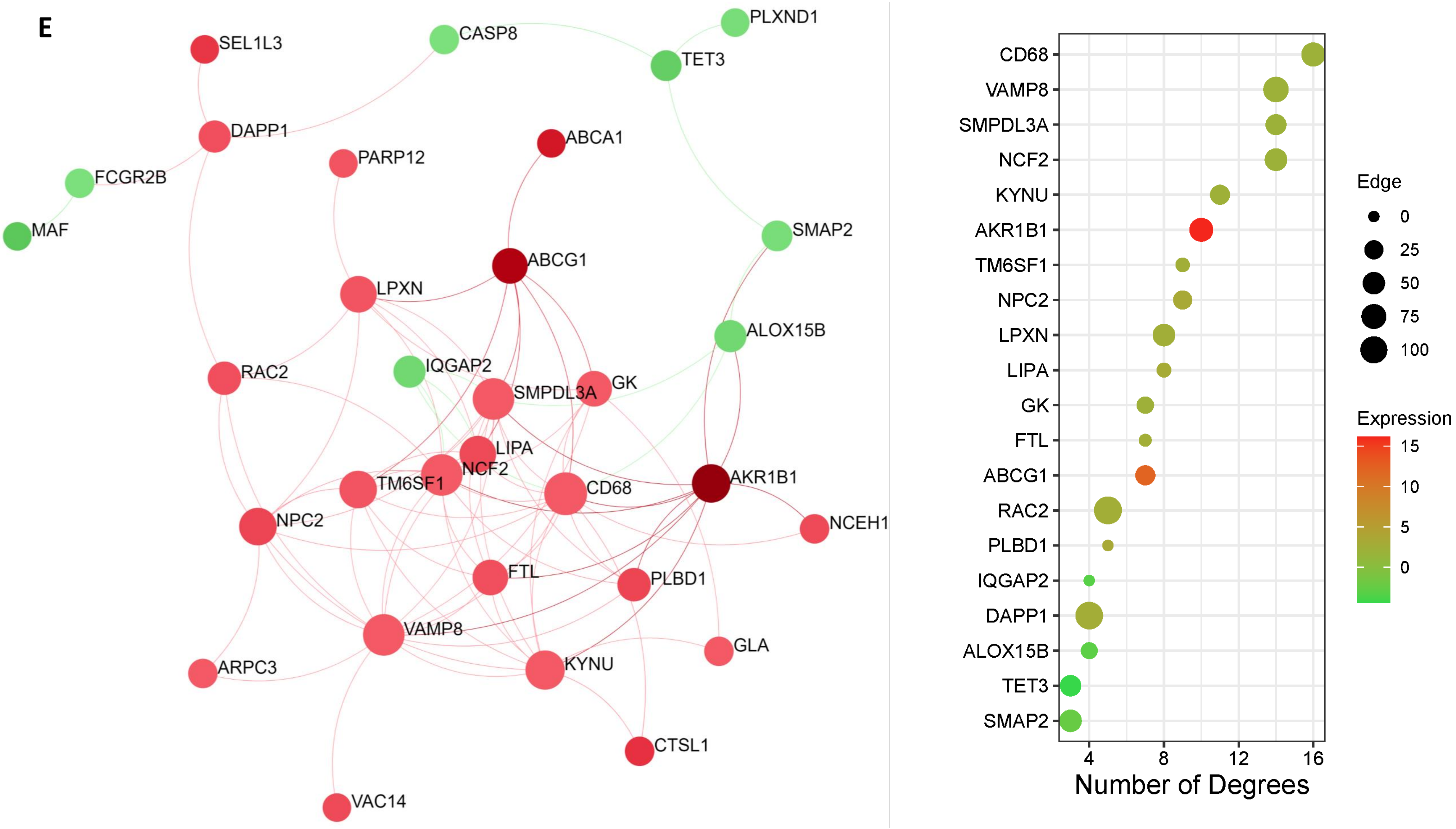
SEL1L3
CASP8
PLXND1
TET3
DAPP1
ABCA1
PARP12
FCGR2B
MAF
SMAP2
ABCG1
LPXN
ALOX15B
RAC2
IQGAP2
SMPDL3A
GK
LIPA
NCF2
TM6SF1
CD68
AKR1B1
NPC2
NCEH1
FTL
PLBD1
VAMP8
GLA
ARPC3
KYNU
CTSL1
VAC14
CD68
VAMP8
SMPDL3A
NCF2
KYNU
AKR1B1
TM6SF1
NPC2
LPXN
LIPA
GK
FTL
ABCG1
RAC2
PLBD1
IQGAP2
DAPP1
ALOX15B
TET3
SMAP2
4
8
12
16
Number of Degrees
Edge
0
25
50
75
100
Expression
15
10
5
0

F

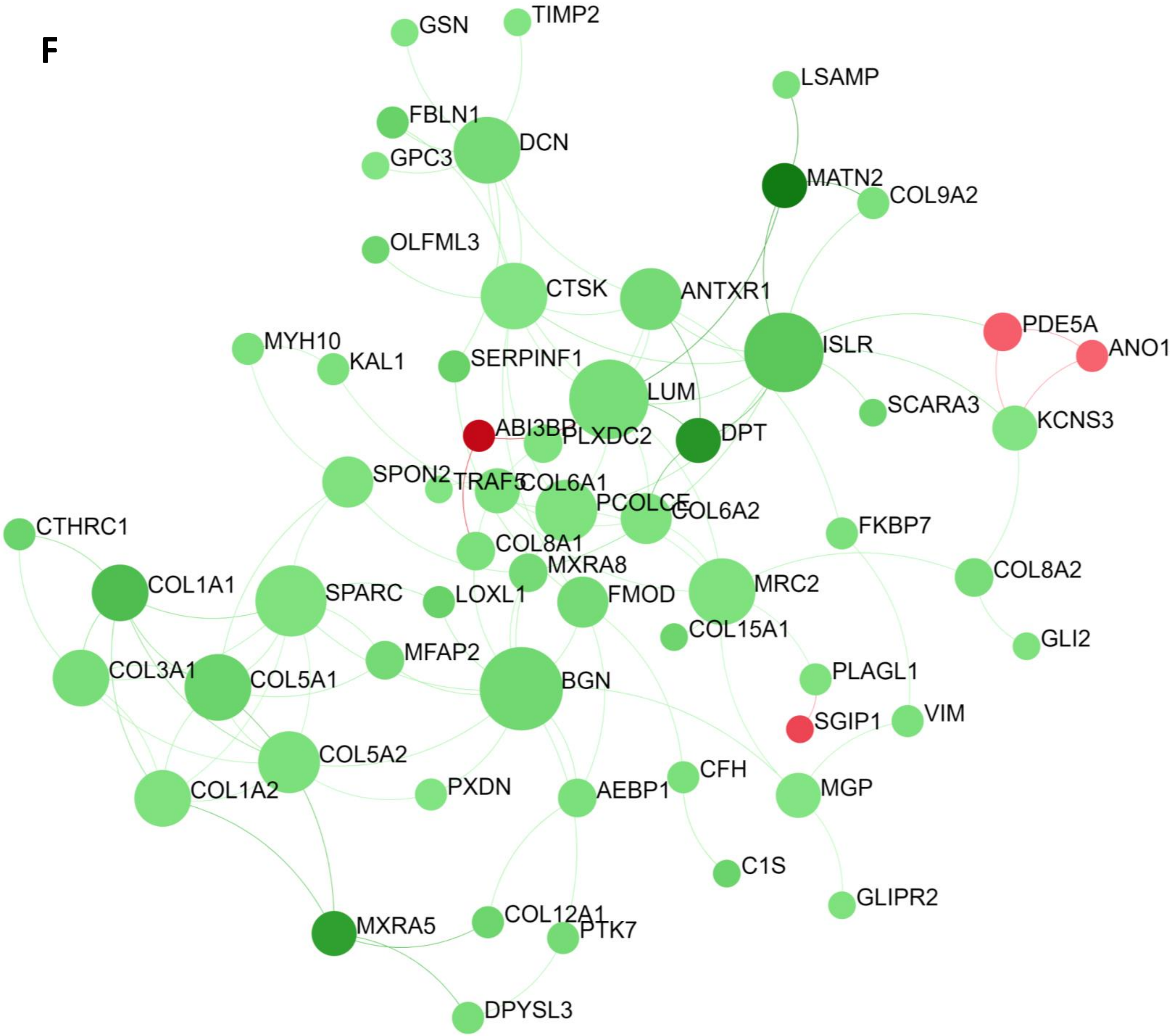
GSN
TIMP2
LSAMP
FBLN1
DCN
GPC3
MATN2
COL9A2
OLFML3
CTSK
ANTXR1
PDE5A
ANO1
MYH10
KAL1
SERPINF1
ISLR
LUM
SCARA3
KCNS3
ABI3BP
PLXDC2
DPT
SPON2
TRAF5
COL6A1
PCOLCE
COL6A2
CTHRC1
FKBP7
COL8A1
MXRA8
COL8A2
COL1A1
SPARC
LOXL1
FMOD
MRC2
COL15A1
GLI2
MFAP2
COL3A1
COL5A1
BGN
PLAGL1
SGIP1
VIM
COL5A2
PXDN
CFH
COL1A2
AEBP1
MGP
C1S
GLIPR2
MXRA5
COL12A1
PTK7
DPYSL3

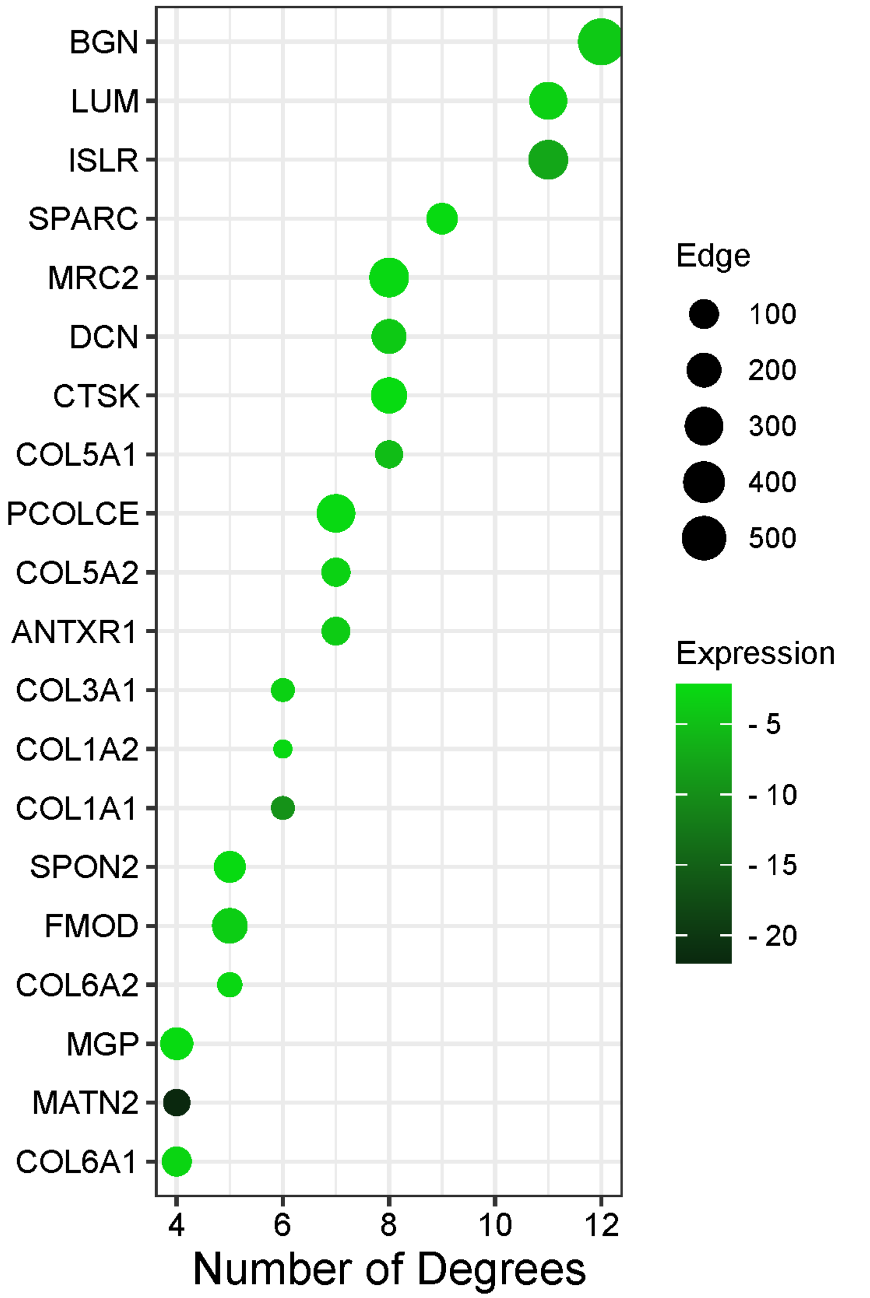
BGN
LUM
ISLR
SPARC
MRC2
DCN
CTSK
COL5A1
PCOLCE
COL5A2
ANTXR1
COL3A1
COL1A2
COL1A1
SPON2
FMOD
COL6A2
MGP
MATN2
COL6A1
4
6
8
10
12
Number of Degrees
Edge
100
200
300
400
500
Expression
- 5
- 10
- 15
- 20

G

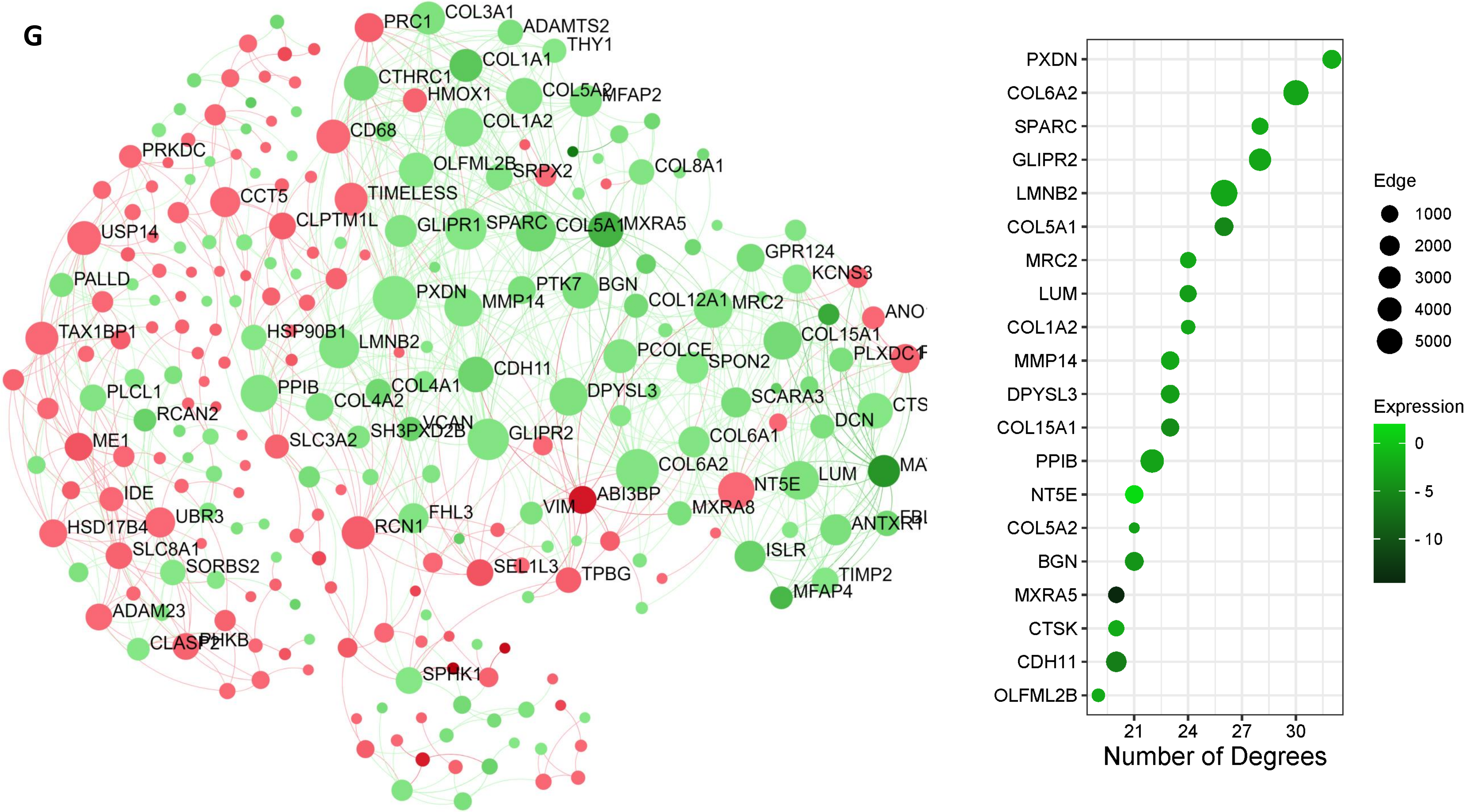

PRC1
COL3A1
ADAMTS2
THY1
COL1A1
CTHRC1
HMOX1
COL5A2
MFAP2
CD68
COL1A2
PRKDC
OLFML2B
SRPX2
COL8A1
CCT5
TIMELESS
CLPTM1L
USP14
GLIPR1
SPARC
COL5A1
MXRA5
GPR124
KCNS3
PALLD
PXDN
PTK7
BGN
MMP14
COL12A1
MRC2
TAX1BP1
HSP90B1
LMNB2
COL15A1
PCOLCE
SPON2
PLXDC1
CDH11
PLCL1
PPIB
COL4A1
DPYSL3
SCARA3
COL4A2
RCAN2
SH3PXD2B
VCAN
GLIPR2
DCN
ME1
SLC3A2
COL6A1
COL6A2
IDE
ABI3BP
NT5E
LUM
UBR3
VIM
MXRA8
HSD17B4
RCN1
FHL3
ANTXR1
SLC8A1
ISLR
SORBS2
SEL1L3
TPBG
TIMP2
MFAP4
ADAM23
CLASP2
PHKB
SPHK1
PXDN
COL6A2
SPARC
GLIPR2
LMNB2
COL5A1
MRC2
LUM
COL1A2
MMP14
DPYSL3
COL15A1
PPIB
NT5E
COL5A2
BGN
MXRA5
CTSK
CDH11
OLFML2B
21
24
27
30
Number of Degrees
Edge
1000
2000
3000
4000
5000
Expression
0
- 5
- 10

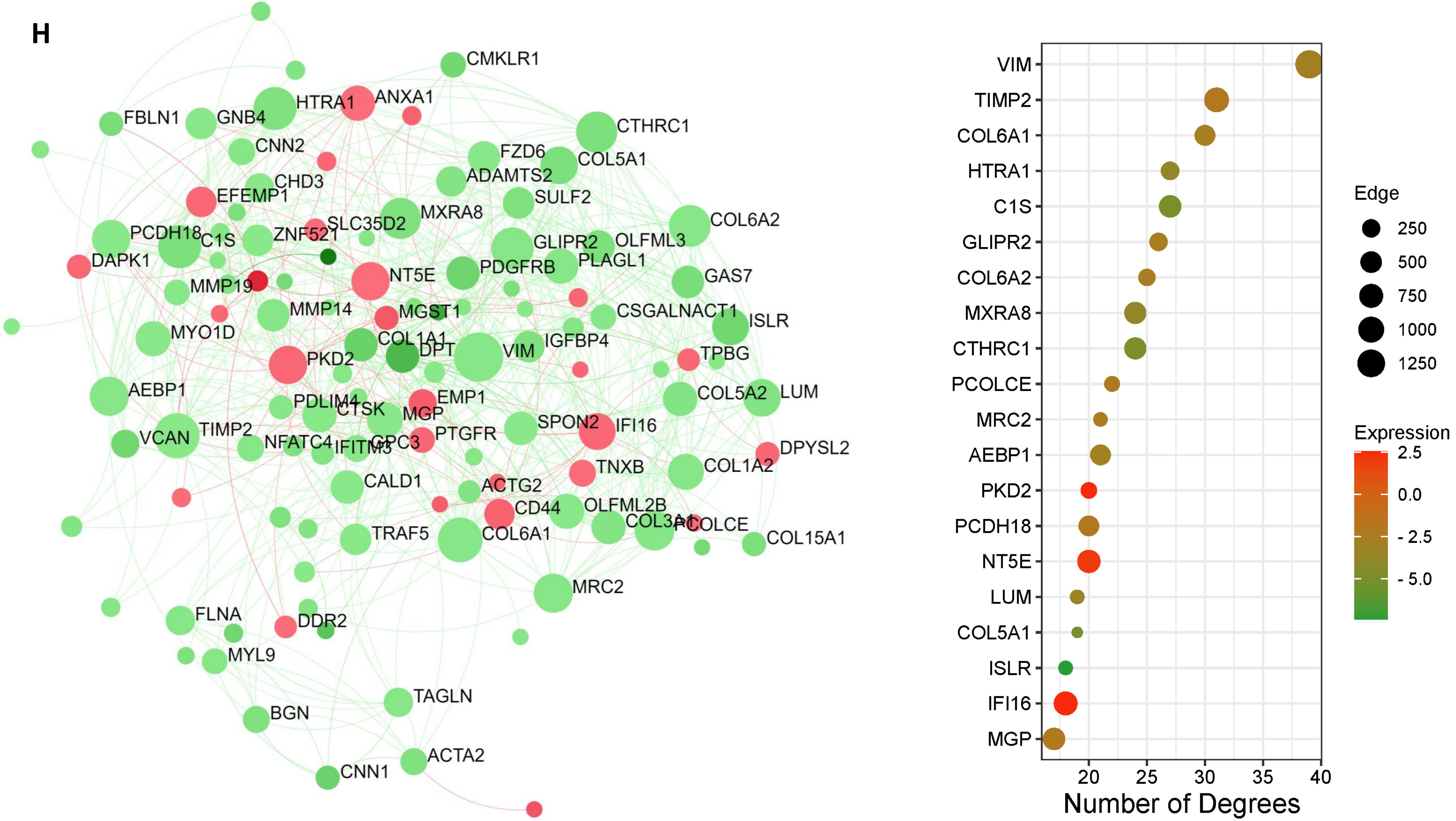

H
CMKLR1
HTRA1
ANXA1
FBLN1
GNB4
CTHRC1
CNN2
FZD6
COL5A1
ADAMTS2
CHD3
EFEMP1
SULF2
MXRA8
SLC35D2
COL6A2
PCDH18
C1S
ZNF521
GLIPR2
OLFML3
DAPK1
PLAGL1
NT5E
PDGFRB
GAS7
MMP19
MMP14
MGST1
CSGALNACT1
ISLR
MYO1D
COL1A1
IGFBP4
DPT
VIM
TPBG
PKD2
AEBP1
COL5A2
LUM
PDLIM4
EMP1
CTSK
MGP
SPON2
IFI16
VCAN
TIMP2
PTGFR
NFATC4
IFITM3
GPC3
DPYSL2
TNXB
COL1A2
CALD1
ACTG2
CD44
OLFML2B
COL3A1
PCOLCE
TRAF5
COL6A1
COL15A1
MRC2
FLNA
DDR2
MYL9
TAGLN
BGN
ACTA2
CNN1
VIM
TIMP2
COL6A1
HTRA1
C1S
GLIPR2
COL6A2
MXRA8
CTHRC1
PCOLCE
MRC2
AEBP1
PKD2
PCDH18
NT5E
LUM
COL5A1
ISLR
IFI16
MGP
20
25
30
35
40
Number of Degrees
Edge
250
500
750
1000
1250
Expression
2.5
0.0
- 2.5
- 5.0

Supplementary Figure S2: PPI interaction networks showing key hub nodes in carnosic acid-treated hAECs. **(A)** Generic,**(B)** whole blood-specific, **(C)** liver-specific, **(D)** adipose tissue-specific, (**E)** coronary artery-specific, **(F)** heart atrium-specific, **(G)** ventricle-specific, and **(H)** skeletal muscle-specific. Each node representing proteins and edges indicating known interactions between two connecting proteins. Red and green nodes denote up and downregulated DEGs, respectively. Figures representing Zero-order network. Bubble plots on the right side of the PPI networks showing top 20 hub genes (seeds) based on number of degrees in each condition. The generic PPI was constructed using the InnateDB Interactome database, and the tissue specific gene coexpression networks were constructed using the tissue/cancer-specific biological networks database
